# Assessing the Quality of Sick Child Care Provided by Community Health Workers

**DOI:** 10.1371/journal.pone.0142010

**Published:** 2015-11-09

**Authors:** Nathan P. Miller, Agbessi Amouzou, Elizabeth Hazel, Tedbabe Degefie, Hailemariam Legesse, Mengistu Tafesse, Luwei Pearson, Robert E. Black, Jennifer Bryce

**Affiliations:** 1 Institute for International Programs, Department of International Health, Johns Hopkins Bloomberg School of Public Health, Baltimore, Maryland, United States of America; 2 United Nations Children’s Fund (UNICEF) Ethiopia Country Office, Addis Ababa, Ethiopia; 3 ABH Services, PLC, Addis Ababa, Ethiopia; California Northstate University College of Medicine, UNITED STATES

## Abstract

**Background:**

As community case management of childhood illness expands in low-income countries, there is a need to assess the quality of care provided by community health workers. This study had the following objectives: 1) examine methods of recruitment of sick children for assessment of quality of care, 2) assess the validity of register review (RR) and direct observation only (DO) compared to direct observation with re-examination (DO+RE), and 3) assess the effect of observation on community health worker performance.

**Methods:**

We conducted a survey to assess the quality of care provided by Ethiopian Health Extension Workers (HEWs). The sample of children was obtained through spontaneous consultation, HEW mobilization, or recruitment by the survey team. We assessed patient characteristics by recruitment method. Estimates of indicators of quality of care obtained using RR and DO were compared to gold standard estimates obtained through DO+RE. Sensitivity, specificity, and the area under receiver operator characteristic curve (AUC) were calculated to assess the validity of RR and DO. To assess the Hawthorne effect, we compared estimates from RR for children who were observed by the survey team to estimates from RR for children who were not observed by the survey team.

**Results:**

Participants included 137 HEWs and 257 sick children in 103 health posts, plus 544 children from patient registers. Children mobilized by HEWs had the highest proportion of severe illness (27%). Indicators of quality of care from RR and DO had high sensitivity for most indicators, but specificity was low. The AUC for different indicators from RR ranged from 0.47 to 0.76, with only one indicator above 0.75. The AUC of indicators from DO ranged from 0.54 to 1.0, with three indicators above 0.75. The differences between estimates of correct care for observed versus not observed children were small.

**Conclusions:**

Mobilization by HEWs and recruitment by the survey teams were feasible, but potentially biased, methods of obtaining sick children. Register review and DO underestimated performance errors. Our data suggest that being observed had only a small positive effect on the performance of HEWs.

## Introduction

Community case management of childhood illness (CCM) is promoted in low-income countries to increase access to life-saving therapies and to reduce child mortality (see Box 1 for definitions of key terms) [1,2]. As CCM programs expand, there is a need to assess the quality of care provided by community-based health workers (CHWs) [3]. Direct observation with re-examination (DO+RE) is usually considered to be the gold standard method for assessing health worker quality of care [4–7]. In this method, a data collector silently observes consultations and records details of the health worker’s assessment, classification, treatment, referral, and counseling of the patient and caregiver. Then a second data collector performs a re-examination with the same patient to obtain gold standard classifications and treatments. Information from the observation is compared to the re-examination results to obtain estimates of indicators of quality of care, such as the proportion of children correctly treated. The main advantages of this method are that the patient’s true signs and symptoms are known and the health worker’s actions are verified. However, DO+RE has several drawbacks. It is resource-intensive, requiring multiple skilled data collectors who spend several hours at each location. Long distances and difficult terrain make traveling to the CHWs’ place of work difficult and expensive [2]. Moreover, the Hawthorne effect, where health workers perform better than under normal circumstances because they are being observed, has been documented [8–11]. Furthermore, caseloads of sick children seen by CHWs are often small [10,12], making it difficult to attain an adequate sample of children. Finally, some children may be managed by CHWs in their home, rather than at a fixed health post. Because of these limitations of DO+RE, alternative methods of recruiting sick children for observation and of assessing quality of care of CHWs need to be developed and assessed.

**Box 1. Definitions of key terms.**

**Community case management of childhood illness (CCM)**: Management of at least one common childhood illness by a community-based health worker.

**Correct classification**: All HEW classifications matched gold standard classifications.

**Correct management**: All HEW treatments matched gold standard treatments including correct dose, duration, and frequency and HEW referral matched the gold standard classification for referral.

**Correct treatment**: All HEW treatments matched gold standard treatments including correct dose, duration, and frequency.

**Eligible iCCM illness**: Lethargy or unconsciousness, convulsions, not eating or drinking, fever/malaria, cough, fast/difficulty breathing, diarrhea, vomiting, ear problem, signs/history of measles, malnutrition, feeding problems, or anemia.

**Integrated community case management of childhood illness (iCCM)**: In general, iCCM refers to the concurrent management of more than one common childhood illness in the community. ICCM in Ethiopia is integrated management by an HEW at the community level of all of the following childhood illnesses: pneumonia, diarrhea, malaria, malnutrition, measles, anemia, and ear infection.

**Major iCCM illnesses**: Pneumonia, diarrhea, malaria, measles, malnutrition, and danger signs.

**Quality of care**: We assessed quality based on whether HEWs correctly assessed, classified, treated, and referred children with iCCM illnesses and provided counseling to caregivers based on Ethiopia iCCM clinical guidelines.

**Sensitivity**: The proportion of children who were correctly managed or received a given clinical action according to the gold standard, who were categorized as having been correctly managed or having received the clinical action according to RR or DO.

**Severe illness**: Any general danger sign (not able to drink/breastfeed, vomits everything, convulsions, lethargic or unconscious), severe pneumonia, diarrhea with severe dehydration, severe persistent diarrhea, persistent diarrhea, dysentery, very severe febrile disease, severe complicated measles, severe complicated malnutrition, or severe anemia.

**Specificity**: The proportion of children who were not correctly managed or did not receive a given clinical action according to the gold standard, who were categorized as being incorrectly managed or not having received the clinical action according to RR or DO.

**Uncomplicated illness**: Uncomplicated pneumonia, diarrhea, malaria, measles, malnutrition, eye infection, or anemia.

**Validity**: The degree to which a method is able to depict the technical quality of services accurately.

Reviewing data in patient registers is a common method for assessing health worker performance due to its feasibility and relatively low cost [10,13–16]. A large number of records can be reviewed quickly and a large sample of records of children with severe or rare illnesses can be attained [5]. Register review (RR) also allows for assessment of routine performance without potential bias caused by the Hawthorne effect. Perhaps the greatest advantage of RR is that it can be conducted as part of routine supervision visits, as long as appropriate procedures are in place to ensure objective reporting of results. However, patient registers often have insufficient data, are incomplete, or are not used at all [5]. Additionally, registers may not reflect the actual practices of the health worker and health workers must be literate enough to fill them properly.

Direct observation (DO) without a separate re-examination offers the benefit of verification of health worker actions without the need for a second data collector. Although this method requires travel to the CHW’s place of work and a sample of sick children, DO can be conducted by a supervisor as part of routine visits if sick children can be found for observation.

Ethiopia scaled up integrated community case management of childhood illness (iCCM) in most regions of the country in 2011 and 2012. ICCM is implemented nationally by Health Extension Workers (HEWs), who provide clinical care in community-based health posts. HEWs are literate salaried government employees and receive one year of pre-service training. The iCCM program provided a six-day training for HEWs on iCCM, supportive supervision, improved supply chain management for essential commodities, and enhanced monitoring and evaluation to enable HEWs to manage childhood pneumonia, diarrhea, malaria, malnutrition, measles, anemia, and ear infection. Children with severe illness are referred to a higher-level health facility.

Through the iCCM program, HEWs are equipped with patient registers that closely follow iCCM clinical guidelines, with spaces for registration of patient information, signs and symptoms, results of diagnostic tests, classification, treatment, referral, counseling, and follow-up. The Ethiopia iCCM sick child register is presented in S1 Fig. These registers were designed to record information on each step in the iCCM algorithms and provide relatively comprehensive information about patients’ signs and symptoms and the decisions and actions taken by the HEW. HEWs record consultations conducted in the health posts as well as home-based consultations in the iCCM register. Because of the relatively high level of literacy of HEWs, and the intensive training and supervision they receive, data from patient registers completed by HEWs is likely to be of higher quality than that in many other community-based healthcare contexts.

Few studies have compared RR or DO to DO+RE for assessing health worker quality of care in low-income countries [4,5], and we are aware of only one study that has compared these methods for assessing CCM [17]. We conducted an assessment of the quality of sick child care provided by HEWs [12]. The assessment provided the opportunity to examine and compare methods for assessing the quality of care provided by HEWs. This study had three objectives: 1) to develop and assess alternative methods of recruitment of sick children for observation in a community setting with low patient volume; 2) to assess the validity of RR and DO compared to DO+RE for assessing HEW quality of care; and 3) to assess the effect of observation on HEW performance.

## Methods

The survey was carried out in May and June 2012, which was just before the start of the malaria transmission season. We conducted a cross-sectional survey in 104 health posts—randomly selected from a total of 490 rural health posts—that were implementing iCCM in Jimma and West Hararghe Zones of Oromia Region, Ethiopia. The sample size was determined assuming proportions of the indicators of interest were 50%, confidence at 95%, 5% non-response, and a design effect of 1.3. This sample size was expected to give a precision for health post-level indicators of ±10 percentage points. Assuming at least two sick children observed per health post, the precision for patient-level indicators would be at least ±9 percentage points.

Oromia is the largest of Ethiopia’s regions, with approximately 30 million people [18]. Eighty-three percent of the population lives in rural areas and most people are subsistence farmers [19]. Malaria risk varied in the study areas, with about 25% of surveyed health posts in high malaria risk areas, 50% in low risk areas, and 25% with no malaria risk.

We employed six survey teams, which were composed of one supervisor, one observer, and one re-examiner, all of whom were health professionals who had worked as iCCM trainers or supervisors. Data collectors were not deployed to areas where they normally work to avoid biasing the results. After seven days of training, all observers and re-examiners achieved at least 90% concordance with gold standard clinicians on three consecutive role-play exams that simulated observation of HEW consultations and re-examinations.

All HEWs present and providing case management services in selected health posts were included. Children had to meet the following inclusion criteria: 1) two to 59 months of age, 2) having at least one complaint consistent with an eligible iCCM illness, and 3) initial consultation for the current illness episode. Children younger than two months of age were excluded because we expected an extremely small sample of children in this age group, which would not justify the extra expense of training data collectors on the algorithm for children younger than two months of age.

The survey instruments (S1 Document) and primary indicators of quality of care were adapted from the WHO Health Facility Survey tool [20], a survey of Health Surveillance Assistants in Malawi [21], and the CCM Global Indicators [22]. The instruments for observation of consultations and re-examination of sick children were based on the Ethiopia iCCM clinical algorithms that HEWs are trained with and that are the basis of the clinical job aids for HEWs. The gold standard classifications from re-examination were based on the same clinical algorithms. Determination of correct management from RR and DO were also based on the iCCM clinical algorithms.

Low patient volume in health posts prompted us to develop alternative methods of recruiting sick children for the assessment. HEWs were notified of upcoming survey visits and were asked to mobilize caregivers to bring sick children to the health post on the day of the visit. If fewer than two children presented at the health post within the first hour of operation, the team supervisor, along with an HEW or community volunteer, recruited sick children from households in the surrounding area. Recruitment was done through door-to-door inquiries among households known to have children under five. If no sick children were present, the household members were asked if they knew of any other sick children in nearby households. The resulting sample of children was obtained through one of three recruitment methods: 1) spontaneous consultation, 2) HEW mobilization, or 3) recruitment by the survey team. Before each consultation, caregivers of sick children were asked to report which recruitment method brought them to the health post.

Survey teams spent one day in each health post collecting data. Gold standard measures of quality of care were obtained through DO+RE. The observer silently observed consultations and noted the assessment tasks carried out by the HEW and the HEW’s classification and prescribed treatments. Then the re-examiner conducted an examination of each child to obtain the gold standard classification. Following the observations and re-examinations, data collectors (observers and re-examiners) extracted information on sick child consultations from iCCM patient registers at all surveyed health posts. They recorded information from iCCM registers using a data collection tool that mirrored the registers (S1 Fig). Data were collected from registers for the same children who were included in DO+RE on the day of data collection plus the last three children aged 2–59 months who were seen by HEWs prior to the day of data collection. Patients who were seen prior to data collection arrived spontaneously for care. Data were entered directly into tablet computers using Open Data Kit (ODK) [23] as the data capture software and were stored in a Research Electronic Data Capture (REDCap) database [24].

For the examination of methods of recruiting sick children, we compared distributions of patient demographic characteristics, illness classifications, and severe illness, and corresponding 95% confidence intervals, stratified by recruitment method.

For the assessment of the validity of RR and DO, we calculated estimates of indicators of quality of care based on DO+RE, RR, and DO for the same children (children observed by the survey team). The objective of this analysis was to compare the estimates obtained had we collected data using RR only or DO only compared to using the gold standard method of DO+RE.

For RR, correct management was determined by checking consistency between recorded signs and symptoms, results of diagnostic tests, treatment, and referral in the patient register. In the register (S1 Fig), the presence of signs and symptoms was recorded by HEWs by circling the name of the sign/symptom that was present. If the sign/symptom was not present, the space was left blank. Therefore, we treated blank signs and symptoms in the register as not present.

One observation was done for each child, and this data was used for both DO+RE and for DO. The difference was that DO did not take into account the data from the re-examination by the survey team member. For DO, estimates of indicators of quality of care were based on consistency between the HEW’s classification of a child and the treatment the child received. Because observers did not record the presence or absence of various signs and symptoms during the observation, we could not base the estimates from DO on consistency between signs and symptoms and treatment, as was done for RR.

We calculated the sensitivity, specificity, and the area under receiver operator characteristic curve (AUC) for RR and for DO, considering DO+RE to be the gold standard. The receiver operator characteristic curve is produced by plotting the sensitivity of a test against 1 –specificity of the test. The area under the curve quantifies the overall ability of a test to discriminate between positive and negative test subjects (in this case children managed correctly and children managed incorrectly) [25]. The AUC was calculated using the sample of children who were determined to have a given condition based on the gold standard re-examination. An AUC of 1.0 represents a perfect test, while an AUC of 0.5 is equivalent to a random guess. There is no widely accepted standard for determining what score signifies an acceptable test, but for the sake of discussion, we will consider an AUC of 0.75 or higher to be acceptable.

To assess the influence of the Hawthorne effect, we compared estimates of indicators of quality of care from RR for children who were observed by the survey team to estimates from RR for children who were not observed by the survey team (i.e. children who received consultations by the HEWs prior to the survey visit). We calculated the arithmetic differences in point estimates of indicators of quality of care between observed and not observed children. Finally, we tested for a significant difference by examining the confidence intervals of the differences between groups of children (whether the confidence intervals included zero) and by calculating p-values for two-sample tests of difference in proportions. Standard errors and associated 95% confidence intervals for point estimates were calculated using the Taylor linearization method to account for clustering of children within health posts [26]. All analyses were carried out in Stata 12 [27].

Ethical approval was obtained from the Institutional Review Boards of the Oromia Regional Health Bureau and the Johns Hopkins University Bloomberg School of Public Health. We obtained informed oral consent from all participating HEWs and caregivers of sick children. Written consent was not obtained because many of the study participants were illiterate. This decision was approved by both IRBs and oral consent was recorded by data collectors on consent forms.

## Results

Of the 104 selected health posts, one was not surveyed because it was closed indefinitely. In the remaining 103 health posts, teams surveyed 137 HEWs and observed and re-examined 257 sick children.

### Characteristics of sick children by recruitment method

Table 1 presents the characteristics and gold standard disease classifications for sick children by recruitment method. Spontaneous consultations accounted for only 18% of the final sample. Another 37% were mobilized by the HEWs and active recruitment of sick children in the community by the survey team accounted for 45% of the sample. Diarrhea and malnutrition were most common among children mobilized by HEWs, and children presenting spontaneously had the highest proportion of pneumonia. Children mobilized by HEWs had the highest proportion of multiple classifications (41.5%) and severe illness (27%). Severe illness was classified in 11% of children presenting spontaneously and in 6% of those recruited by the survey team. The proportion with severe illness was significantly higher among mobilized children than among children recruited by survey teams.

**Table 1:** Characteristics of the sample of sick children by recruitment method.

Characteristic	Total	Spontaneous	Mobilized by HEWs	Recruited by survey team
	N = 257	N = 45	N = 96	N = 116
	n (%)	n (%)	n (%)	n (%)
**Zone**				
Jimma	152 (59.1)	32 (71.1)	39 (40.6)	81 (69.8)
W. Hararghe	105 (40.9)	13 (28.9)	57 (59.4)	35 (30.2)
**Age**				
2–11 months	94 (36.6)	17 (37.8)	34 (35.4)	43 (37.1)
12–23 months	92 (35.8)	17 (37.8)	34 (35.4)	41 (35.3)
24–35 months	39 (15.2)	5 (11.1)	21 (21.9)	13 (11.2)
36–47 months	22 (8.6)	5 (11.1)	4 (4.2)	13 (11.2)
48–59 months	10 (3.9)	1 (2.2)	3 (3.1)	6 (5.2)
**Sex**				
Male	129 (50.2)	17 (37.8)	51 (53.1)	61 (52.6)
Female	128 (49.8)	28 (62.2)	45 (46.9)	55 (47.4)
**Disease classification**				
Pneumonia	39 (15.2)	10 (22.2)	11 (11.5)	18 (15.5)
Diarrhea	169 (65.8)	24 (53.3)	73 (76.0)	72 (62.1)
Malaria	3 (1.2)	1 (2.2)	1 (1.0)	1 (0.9)
Measles	5 (2.0)	1 (2.2)	0 (0.0)	4 (3.5)
Malnutrition	32 (12.5)	5 (11.1)	19 (19.8)	8 (6.9)
>1 classification	74 (28.8)	10 (21.2)	40 (41.7)	24 (20.7)
Severe illness	38 (14.8)	5 (11.1)	26 (27.1)	7 (6.0)

### Validity of register review and DO

Eleven of the 257 observed children were missing from the patient registers or had insufficient data, so they were excluded from this analysis, giving a final sample of 246 children. The point estimates, sensitivity, specificity, and AUC for indicators of quality of care from RR and DO compared to DO+RE are shown in Table 2. Results for children with malaria and measles are not shown because of small sample sizes.

**Table 2:** Point estimates, sensitivity, specificity, and area under receiver operator characteristic curve for indicators of quality of care from register review and direct observation only compared to direct observation with re-examination.

Indicator	DO+RE^1^		RR^2^						DO^3^					
	n	% of children	n	% of children	Sens.	Spec.	n for AUC analysis	AUC^5^	n	% of children	Sens.	Spec.	n for AUC analysis	AUC
		(95% CI)^4^		(95% CI)						(95% CI)				
Correct treatment of pneumonia	37	73.0	42	78.6	92.6	10.0	37	0.51	38	92.1	100.0	30.0	37	0.65
		(56.6, 84.8)		(58.1, 90.7)				(0.4, 0.62)		(76.8, 97.6)				(0.5, 0.8)
Correct treatment of diarrhea	163	78.5	160	73.8	84.4	68.6	163	0.76	163	71.8	89.8	91.4	163	0.9
		(70.6, 84.8)		(64.4, 81.4)				(0.68, 0.85)		(63.5, 78.8)				(0.85, 0.96)
Correct treatment of malnutrition	31	61.3	32	71.9	68.4	66.7	31	0.68	22	45.5	36.8	75.0	31	0.56
		(41.7, 77.8)		(51.2, 86.2)				(0.5, 0.85)		(22.8, 70.2)				(0.39, 0.73)
Correct management of major iCCM illnesses	246	63.4	246	65.9	83.3	64.4	246	0.74	246	76.42	96.8	58.9	246	0.78
		(56.1, 70.2)		(58.4, 72.6)				(0.68, 0.8)		(70.2, 81.7)				(0.73, 0.83)
Correct management of severe illness	37	32.4	40	32.5	50.0	44.0	37	0.47	34	41.2	83.3	48.0	37	0.66
		(20.0, 48.0)		(17.5, 52.3)				(0.29, 0.65)		(26.5, 57.6)				(0.51, 0.81)
Correct dose, duration, and schedule for all treatments	165	92.1	167	91.6	94.1	38.5	165	0.67	246	94.7	100.0	100.0	165	1.0
		(86.5, 95.5)		(85.5, 95.3)				(0.52, 0.8)		(90.9, 97.0)				-
Unnecessary antibiotic or antimalarial prescribed	246	5.3	246	3.7	38.5	98.3	246	0.68	246	2.4	38.5	99.6	246	0.69
		(2.8, 9.7)		(1.6, 8.2)				(0.55, 0.82)		(1.0, 5.9)				(0.55, 0.83)
Vitamin A supplement given when needed	64	18.8	49	20.4	75.0	51.9	64	0.63	56	23.2	91.7	55.8	64	0.74
		(10.2, 31.8)		(9.5, 38.5)				(0.49, 0.78)		(12.4, 39.2)				(0.63, 0.84)
Mebendazole given when needed	29	20.7	23	17.4	66.7	52.2	29	0.6	27	22.2	83.3	65.2	29	0.74
		(9.3, 39.9)		(6.2, 40.3)				(0.36, 0.83)		(9.3, 44.2)				(0.55, 0.93)
Correct referral when needed	61	52.5	50	66.0	75.0	31.0	61	0.53	44	81.8	93.8	13.8	61	0.54
		(39.0, 65.5)		(48.2, 80.2)				(0.42, 0.64)		(66.9, 90.9)				(0.46, 0.61)
Correct classification of major iCCM illnesses	246	52.9	246	67.9	86.2	52.6	246	0.69	N/A					
		(45.4, 60.2)		(60.9, 74.2)				(0.63, 0.75)						
Immunization status checked^6^	88	97.7	88	95.5	93.0	0.0	88	0.47	N/A					
		(91.1, 99.5)		(88.5, 98.3)				(0.44, 0.49)						

^1^ Direct observation with re-examination.

^2^ Register review.

^3^ Direct observation.

^4^ Indicators of quality of care from DO+RE were previously published in Miller et al.[12].

^5^ Area under receiver operator characteristic curve.

^6^ Children under 12 months of age.

Sensitivity of RR was reasonably high (≥75%) for eight of the 12 indicators. However, specificity was below 70% for 11 indicators. The AUC ranged from 0.47 to 0.76 for the indicators assessed. Register review did a poor job at detecting performance errors for all indicators (Note that the low sensitivity and high specificity for the indicator of “Unnecessary antibiotic or antimalarial prescribed” indicates underestimation of errors [provision of unnecessary treatment] and correct identification of good performance). The summary indicator of correct management of major iCCM illnesses had a sensitivity of 83%, specificity of 64%, and AUC of 0.74, which indicates overall fair agreement, but with underestimation of treatment errors.

Sensitivity of DO was ≥75% for eight of 10 indicators assessed. Correct classification could not be assessed for DO because observers did not record presence or absence of signs and symptoms and assessment of immunization status was not included because direct observation is the source of information on assessment tasks performed for DO and DO+RE, so the results are exactly the same. Specificity was ≥75% for four indicators and was below 70% for six indicators. The AUC for indicators from DO was between 0.54 and 1.0. For the indicator of correct management of major iCCM illnesses, sensitivity was 97%, specificity was 59%, and the AUC was 0.78.

### Hawthorne effect

We abstracted data for 544 children from iCCM patient registers. Of these children, 246 were observed by the survey team and 298 were seen by HEWs prior to the day of the survey visit. Table 3 shows the estimates of indicators of quality of care from RR for children who were observed and children not observed, the difference between the two estimates, and the p-value of the test of difference. The differences between the two estimates were relatively small for most of the indicators and the difference was borderline significant for only one indicator. For the summary indicator of correct management of major iCCM illnesses, the estimates were similar (66% of observed children compared to 68% for children not observed, p = 0.64) for both groups of children.

**Table 3:** Differences in estimates of indicators of quality of care from register review for children observed by the survey team and children not observed.

Indicator	Observed by survey team		Not observed by survey team		Difference	p-value
	n	% (95% CI)	n	% (95% CI)	(95% CI)	
Correct classification of major iCCM illnesses	246	67.9 (60.9, 74.2)	298	73.2 (66.4, 79.0)	-5.3 (-13.0, 2.4)	0.18
Correct treatment of pneumonia	42	78.6 (58.1, 90.7)	52	84.6 (71.4, 92.4)	-6.0 (-21.8, 9.8)	0.45
Correct treatment of diarrhea	160	73.8 (64.4, 81.4)	155	68.4 (59.0, 76.5)	5.4 (-4.6, 15.4)	0.29
Correct treatment of malnutrition	32	71.9 (51.2, 86.2)	51	62.8 (43.5, 78.6)	9.1 (-11.4, 29.6)	0.39
Correct management of major iCCM illnesses	246	65.9 (58.4, 72.6)	298	67.8 (60.9, 74.0)	-1.9 (-9.9, 6.1)	0.64
Correct management of severe illness	40	32.5 (17.5, 52.3)	26	23.1 (10.9, 42.4)	9.4 (-12.4, 31.2)	0.41
Correct dose, schedule, and duration for all treatments	167	91.6 (85.5, 95.3)	210	89.5 (85.0, 92.8)	2.1 (-3.8, 8.0)	0.49
Unnecessary antibiotic or antimalarial prescribed	246	3.7 (1.6, 8.2)	298	5.0 (2.9, 8.6)	-1.3 (-4.7, 2.1)	0.46
Immunization status checked^1^	88	95.5 (88.5, 98.3)	89	97.8 (91.0, 99.5)	-2.3 (-7.6, 3.0)	0.4
Vitamin A supplement given when needed	49	20.4 (9.5, 38.5)	59	15.3 (5.9, 34.2)	5.1 (-9.4, 19.6)	0.49
Mebendazole given when needed	23	17.4 (6.2, 40.3)	36	2.8 (0.3, 19.6)	14.6 (-1.8, 31.0)	0.05
Correct referral when needed	50	66.0 (48.2, 80.2)	32	56.3 (38.9, 72.2)	9.7 (-11.9, 31.3)	0.38

^1^ Children under 12 months of age.

## Discussion

### Recruitment of sick children

Mobilization of sick children by HEWs and recruitment by the survey team each provided more than twice as many sick children than did spontaneous consultations. Survey teams were able to contact HEWs using cellular phones or by passing the message through local administrators, and HEWs usually complied with the mobilization request. Active recruitment of sick children from the community also proved to be easier and more productive than we anticipated.

The highest proportion of children with severe illness was found in the children mobilized by the HEWs. One explanation may be that the HEWs are familiar with their communities, and may have mobilized in households where they knew there were very ill children. Other observed differences in child characteristics may also be due to HEWs and/or survey teams introducing implicit selection criteria. These results suggest that mobilization by HEWs and recruitment of sick children by survey teams are feasible methods of obtaining relatively large samples of sick children, including children with severe illness. However, differences were observed between the groups of children, which indicate the potential for bias associated with active mobilization or recruitment. The small sample sizes of children within each group make it difficult to assess these differences with a high degree of precision.

### Validity of register review and DO

Our assessment of the validity of RR and DO, compared to DO+RE, found that sensitivity was high for the majority of indicators, but specificity was low for both RR and DO. High sensitivity and low specificity indicate that RR and DO were reasonably good at identifying correct practices, but less useful for identifying errors in practice. The AUC for RR and DO varied widely between indicators, ranging from very poor to good validity, but these methods did not perform well for the majority of indicators.

Our results are generally consistent with previous assessments of RR that showed low to moderate validity of RR [4,5,17]. The only previous assessment of RR in a CHW setting found that RR provided better estimates for management of diarrhea than for fast breathing and severe illness [17], which is also consistent with our findings. On the other hand, our results diverge with those of another study in health facilities that found that RR was more useful for identifying performance errors than for identifying correct performance [4]. Given the feasibility and relative low cost of RR compared to DO+RE, RR will continued to be used for routine monitoring purposes. These results suggest that RR will likely overestimate performance and should be used with caution. Estimates of quality of care from RR should be interpreted keeping in mind that the estimates may underestimate performance errors. Given the relatively low consistency with the gold standard estimates, it is not recommended to use RR for assessing quality of care for evaluation purposes. Register review may also be less useful in other developing country contexts where data quality and completeness in patient registers are lower than in Ethiopia. More work is needed to determine how the quality of data in patient registers can be improved in a CHW setting.

Our results correspond with previous results that show that DO overestimates the proportion of cases correctly managed by CHWs by around 13–14% [17]. To further enhance the validity of results obtained through DO, data collectors should record the presence or absence of signs and symptoms as determined by the observer. If information is not available from the observation (i.e. the HEW does not ask about a given sign or symptom or the caregiver does give a clear answer), the observer could ask the necessary questions or perform the needed examinations or tests to determine the presence or absence of the signs and symptoms and the child’s gold standard classification. If validity were found to be reasonably high using this method, it could provide the advantage of measuring actual performance of health workers, while eliminating the need for multiple data collectors. Direct observation, combined with the sick child recruitment techniques described above, could be conducted during routine supervision visits, potentially providing a valuable compromise between the need for a high degree of rigor and lower cost.

### Hawthorne effect

The comparison of estimates of indicators of quality of care from RR for children who were observed by the survey team to estimates for children who were not observed found that most of the differences were small. Of the four indicators with larger differences (above nine percentage points), estimates were higher for children who were observed. All but one of these differences was non-significant, but this may be due to small sample sizes. However, all of the indicators with relatively large sample sizes showed small, non-significant differences. These data suggest that the effect of observation in this setting was small.

Previous assessments of the effect of observation on quality of care in developing countries found that health workers performed substantially better when under observation. However, those studies often assessed the affect of observation using inconsistent methods (e.g. observation versus simulated client [11] or hospital-based observation versus RR in communities [10], or observation versus exit interview [8]). It is not clear that differences seen in these studies are entirely due to the Hawthorne effect rather than inconsistencies in the data collection methods. By using RR to obtain estimates of performance for observed and not observed children, we attempted to eliminate bias caused by inconsistency of methods.

### Limitations

These analyses have several limitations. First, small sample sizes for some indicators limited our ability to assess management of malaria and measles and to detect statistically significant differences between groups. Second, it is possible there was misclassification regarding whether children were obtained through spontaneous consultations or HEW mobilization. Third, patients recruited through HEW mobilization or recruitment by the survey team may have been different from typical patients seen by HEWs who arrive spontaneously. This could have biased the assessment of the Hawthorne effect, which compared the two different groups of children. Fourth, it is possible that signs or symptoms that were left blank in the patient register were actually present and the HEW failed to record these, which would have led us to mistakenly assume the sign/symptom was not present. This could have caused incorrect assessments of correct care from RR. Finally, the failure to record presence or absence of signs and symptoms of sick children during direct observation made it impossible to obtain a gold standard classification with which to compare the HEWs classification and treatment from DO, and therefore greatly limited the usefulness of the estimates of indicators of quality of care from DO.

## Conclusions

These analyses support the use of direct observation with re-examination for assessing quality of care of CHWs when possible. Samples of sick children can be obtained through mobilization by CHWs and recruitment of children by survey teams, but bias may be introduced. Our assessment of the validity of RR and DO suggests that RR and DO are not ideal proxies for DO+RE. However, because DO+RE is too costly to be implemented as part of routine monitoring and evaluation activities, program implementers will need more affordable methods of assessing quality of care. The advantages in cost and feasibility of RR encourage its continued use for routine data collection. However, improvements to the quality of data from RR are needed for this method to provide more accurate estimates. Direct observation is a promising method that could offer valid estimates of quality of care at lower cost than DO+RE. Further research is needed to rigorously assess the validity of DO.

## Supporting Information

S1 Fig Ethiopia iCCM sick child register. (JPG)

S1 Document Ethiopia iCCM quality of care and implementation strength survey instruments. (PDF)
