# Supplementary figures and images for: Assessing the Quality of Sick Child Care Provided by Community Health Workers
Source: PLoS One. 2015 Nov 9;10(11):e0142010. doi: 10.1371/journal.pone.0142010 (PMC4638333; doi:10.1371/journal.pone.0142010)

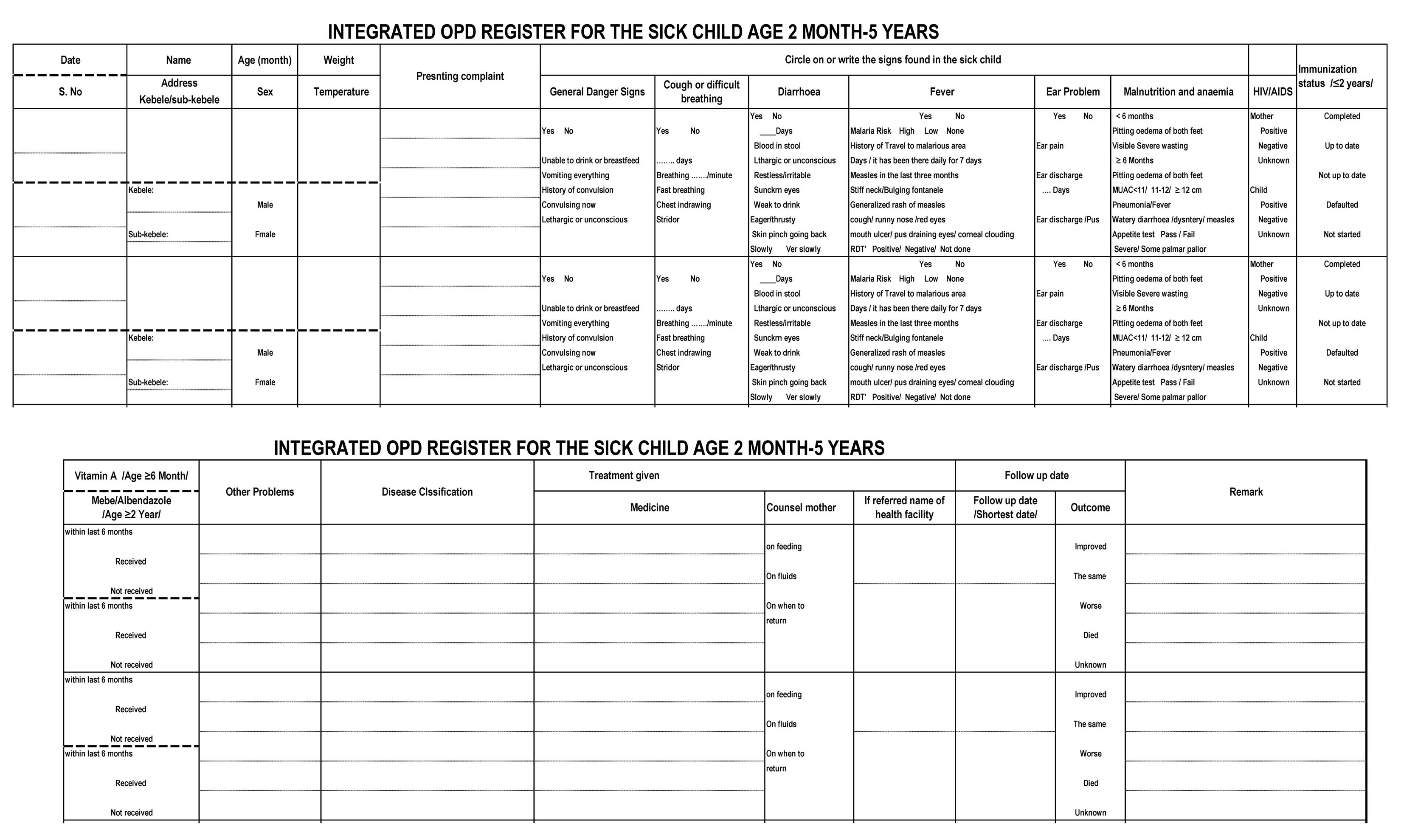

Supplement: S1 Fig — (JPG) [file pone.0142010.s001.jpg]
