# Supplementary material for: Assessing the Quality of Sick Child Care Provided by Community Health Workers
Source: PLoS One. 2015 Nov 9;10(11):e0142010. doi: 10.1371/journal.pone.0142010 (PMC4638333; doi:10.1371/journal.pone.0142010)
Supplement: S1 Document — (PDF) [file pone.0142010.s002.pdf]

INSTITUTE FOR INTERNATIONAL PROGRAMS

Ethiopia iCCM Implementation Snapshot & Quality of Care Assessment

IIP-JHU & ABH Services, PLC

Read the informed consent and obtain agreement from the HEWs.
Fill out one Health Post Questionnaire Panel per health post.

Health Post Questionnaire Panel

HP1.	Zone: ___________________________________
HP2.	Woreda: _________________________________
HP3.	Kebele: _________________________________
HP4.	Health post: _________________________________
HP5.	Distance of health post from nearest referral health location: ______ km
HP6.	Malaria risk of kebele: (1) High (2) Low (3) No malaria
HP7.	Population of children under five in the health post catchment area: ________________

Date of visit: __ __ /__ __ /__ __   Data collector name: ________________   Result: ___

Number of observations of consultations completed: _____   Number of re-examinations completed: _____

Result codes:

1 = Completed
2 = Partially completed
3 = Health post closed / no HEW available
4 = Refused
5 = Health post could not be found
8 = Other, specify: ______________________________

**Supervisor**	**Senior supervisor (quality check)**
Name ___________________	Name ___________________
Date__ __ /__ __ / __ __	Date__ __ / __ __ / __ _

Form 1. Observation Checklist – child (2 – 59 months)

Read informed consent and obtain agreement from child's caretaker.
Then fill out one Observation Checklist per sick child.

Unique child ID: ☐☐☐☐ **Unique HEW ID**: ☐☐☐ Data collector name: ………………

Zone: …………………. Woreda: …………………. Kebele: …………………. Health post: ………………….

HEW number: …………………. HEW name: ………………….

Caretaker number: ……… Caretaker relationship: (1) Biological Mother (2) Father (8) Other: …..………..

Child name: …………………. Child number: ……….. Sex: (1) M (2) F Age (completed months): ………

Consultation type: (1) Spontaneous (2) Mobilized by HEW (3) Recruited by survey team

Time of start of observation: ……… (Ethiopian time)

Interruption time 1: ……… Resumption time 1: ………

Interruption time 2: ……… Resumption time 2: ………

Interruption time 3: ……… Resumption time 3: ………

ASSESSMENT MODULE

Observe silently and do not interfere with the consultation. Record what you hear and see.

A1. What reasons does the caretaker give for bringing the child to the health post?
Circle all signs mentioned.

A.	Fast/difficult breathing	(1) mentioned	(2) not mentioned
B.	Cough	(1) mentioned	(2) not mentioned
C.	Pneumonia	(1) mentioned	(2) not mentioned
D.	Diarrhoea	(1) mentioned	(2) not mentioned
E.	Fever	(1) mentioned	(2) not mentioned
F.	Malaria	(1) mentioned	(2) not mentioned
G.	Convulsions	(1) mentioned	(2) not mentioned
H.	Difficulty drinking or breastfeeding	(1) mentioned	(2) not mentioned
I.	Vomiting	(1) mentioned	(2) not mentioned
J.	Ear problem	(1) mentioned	(2) not mentioned
K.	Other problem, specify:	(1) mentioned	(2) not mentioned
L.	Other problem, specify:	(1) mentioned	(2) not mentioned
M.	Other problem, specify:	(1) mentioned	(2) not mentioned

Does the health worker ask about the following signs and symptoms?
Note: "Avail." means the information was already available. Either the patient (or caretaker) spontaneously offers the information, or the patient has already given the information in response to a previous question, or the patient very obviously has the sign (e.g., convulsions, vomiting, etc.).
Note: The questions below refer to whether the child currently has or has had the given signs/symptoms during the illness episode.

**Weight**		
A2A.<br><br>A2B.	Does the HEW weigh the child?<br><br>What is the child's weight?	(1) Yes<br>(2) No → ***Skip to A3A***<br>……..... kgs
**Danger Signs**		
A3A.	Does the HEW ask if the child is NOT able to drink or breastfeed ANYTHING?	(1) Yes<br>(2) No<br>(3) Avail.
A3B.	Does the HEW **check** if the child is able to drink or breastfeed (*by offering water or breastmilk*)?	(1) Yes<br>(2) No<br>(3) Avail.
A4.	Does the HEW ask if the child is vomiting EVERYTHING?	(1) Yes<br>(2) No<br>(3) Avail.
A5.	Does the HEW ask whether the child has convulsions?	(1) Yes<br>(2) No<br>(3) Avail.
A6.	Does the HEW check for lethargy or unconsciousness (try to wake up the child)?	(1) Yes<br>(2) No<br>(3) Avail.
**Cough/Difficult Breathing**		
A7A.	Does the HEW ask if the child has cough or difficult breathing?	(1) Yes<br>(2) No → ***Skip to A8A***<br>(3) Avail.
A7B.	Does the child have cough or difficult breathing?	(1) Yes<br>(2) No → ***Skip to A8A***
A7C.	Does the HEW ask how long the child has had cough or difficult breathing?	(1) Yes<br>(2) No<br>(3) Avail.
A7D.	Does the HEW count breaths in 1 minute?	(1) Yes<br>(2) No → ***Skip to A7F***
A7E.	How many breaths does the HEW count in 1 minute?	……..... bpm
A7F.	Does the HEW look for chest indrawing?	(1) Yes<br>(2) No<br>(3) Avail.
A7G.	Does the HEW look and listen for stridor?	(1) Yes<br>(2) No<br>(3) Avail.
**Diarrhoea**		
A8A.	Does the HEW ask if the child has diarrhoea (loose stools)?	(1) Yes<br>(2) No → ***Skip to A9A***<br>(3) Avail.

A8B.	Does the child have diarrhoea?	(1) Yes<br>(2) No → ***Skip to A9A***
A8C.	Does the HEW ask how long the child has had diarrhoea?	(1) Yes<br>(2) No<br>(3) Avail.
A8D.	Does the HEW ask if there is blood in the stool?	(1) Yes<br>(2) No<br>(3) Avail.
A8E.	Does the HEW check if the child is restless or irritable?	(1) Yes<br>(2) No<br>(3) Avail.
A8F.	Does the HEW offer the child fluid?	(1) Yes<br>(2) No<br>(3) Avail.
A8G.	Does the HEW pinch the skin of the abdomen?	(1) Yes<br>(2) No
**Fever**		
A9A.	Does the HEW ask or feel for fever (reported or now) or take the child's temperature?	(1) Yes<br>(2) No → ***Skip to A10A***<br>(3) Avail.
A9B.	Does the HEW take the child's temperature?	(1) Yes<br>(2) No → ***Skip to A9D***
A9C.	What is the child's temperature?	…….... degrees
A9D.	Does the child have fever or history of fever (last 48 hours)?	(1) Yes<br>(2) No → ***Skip to A10A***
A9E.	Does the HEW ask how long the child has had fever?	(1) Yes<br>(2) No<br>(3) Avail.
A9F.	Does the HEW ask if the fever has been present every day?	(1) Yes<br>(2) No<br>(3) Avail.
A9G.	Does the HEW check if the child has a stiff neck?	(1) Yes<br>(2) No
A9H.	Does the HEW check if the child has bulged fontanel?	(1) Yes<br>(2) No
A9I.	Does the HEW ask if the child has had measles in the last 3 months **or** check for signs of measles?<br>*Note: Signs of measles defined as generalized rash and one of these: cough, runny nose, red eyes. Observe the child for runny nose and red eyes.*	(1) Yes<br>(2) No → ***Skip to A9N***<br>(3) Avail.
A9J.	Does the HEW check for generalized rash?	(1) Yes<br>(2) No<br>(3) Avail.
A9K.	Does the child have history of measles in the last 3 months **or** signs of measles?	(1) Yes<br>(2) No → ***Skip to A9N***

A9L.	Does the HEW check if the child has mouth ulcers?	(1) Yes<br>(2) No
A9M.	Does the HEW look at the child's eyes for pus or clouding of the cornea?	(1) Yes<br>(2) No<br>(3) Avail.
A9N.	Does the HEW perform an RDT for the child?	(1) Yes<br>(2) No → ***Skip to A10A***
A9O.	What is the result of the RDT?	(1) Pos. falciparum<br>(2) Pos. vivax<br>(3) Pos. falciparum & vivax<br>(4) Neg<br>(5) Invalid
A9P.	Is the RDT performed correctly?	(1) Yes<br>(2) No
**Ear Problem**		
A10A.	Does the HEW ask if the child has an ear problem?	(1) Yes<br>(2) No → ***Skip to A11A***<br>(3) Avail.
A10B.	Does the child have an ear problem?	(1) Yes<br>(2) No → ***Skip to A11A***
A10C.	Does the HEW ask how long the child has had an ear problem?	(1) Yes<br>(2) No<br>(3) Avail.
A10D.	Does the HEW ask if there is ear pain?	(1) Yes<br>(2) No<br>(3) Avail.
A10E.	Does the HEW check if there is ear discharge/pus draining from the ear?	(1) Yes<br>(2) No<br>(3) Avail.
**Malnutrition**		
A11A.	Does the HEW press on both feet to look for swelling?	(1) Yes<br>(2) No
A11B.	Does the HEW look for visible severe wasting?	(1) Yes<br>(2) No<br>(3) Avail.
A11C.	Does the HEW measure the child's MUAC?	(1) Yes<br>(2) No → ***Skip to A11E***
A11D.	What is the child's MUAC measurement (in cm)?	(1) <11<br>(2)11-<12<br>(3) ≥12
A11E.	Does the HEW give the child an appetite test?	(1) Yes<br>(2) No<br>(3) Avail.

**Anaemia**		
A12.	Does the HEW look for palmar pallor?	(1) Yes<br>(2) No
**HIV**		
A13A.	Does the HEW ask about the mother's HIV status?	(1) Yes<br>(2) No<br>(3) Avail.<br>(9) NA
A13B.	Does the HEW ask about the child's HIV status?	(1) Yes<br>(2) No<br>(3) Avail.
**Vaccination**		
A14A.	Does the HEW ask to see the child's vaccination card?	(1) Yes<br>(2) No → ***Skip to A14C***<br>(3) Avail.
A14B.	Is the child's vaccination card available?	(1) Yes<br>(2) No
A14C.	Does the HEW ask the caretaker about the child's vaccination history?	(1) Yes<br>(2) No<br>(3) Avail.
**Vitamin A**		
A15.	Does the HEW ask if the child has received a dose of vitamin A in the previous 6 months?	(1) Yes<br>(2) No<br>(3) Avail.
**Mebendazole/Albendazole**		
A16.	Does the HEW ask if the child has received a dose of mebendazole/albendazole in the previous 6 months?	(1) Yes<br>(2) No<br>(3) Avail.
**Other Problems**		
A17.	Does the HEW ask if there are any other problems?<br>Specify: ……………………………<br>……………………………<br>……………………………	(1) Yes<br>(2) No<br>(3) Avail.

CLASSIFICATION MODULE

Look at the iCCM registration book for the patient's classification. If the classification is not recorded in the registration book, ask the HEW what the child's classifications are. Ask "Any other classification?" until the health worker has stated all classifications. Do not ask for each specific classification.

C1. Does the HEW give one or more classifications for the child?

(1) Yes

(2) No → ***Skip to Treatment Module (T1)***

***Observer** record all classifications below:*		
C2.	One or more general danger signs (unable to drink or breastfeed, vomits everything, convulsions, lethargic/unconscious)	(1) Yes (2) No
C3A.	Severe pneumonia/very severe disease	(1) Yes (2) No
C3B.	Pneumonia	(1) Yes (2) No
C3C.	No pneumonia: cough or cold	(1) Yes (2) No
C4A.	Diarrhoea, severe dehydration	(1) Yes (2) No
C4B.	Diarrhoea, some dehydration	(1) Yes (2) No
C4C.	Diarrhoea, no dehydration	(1) Yes (2) No
C4D.	Severe persistent diarrhoea	(1) Yes (2) No
C4E.	Persistent diarrhoea	(1) Yes (2) No
C4F.	Dysentery	(1) Yes (2) No
C5A.	Very severe febrile disease	(1) Yes (2) No
C5B.	Malaria	(1) Yes (2) No
C5C.	Fever, malaria unlikely	(1) Yes (2) No
C5D.	Fever, no malaria	(1) Yes (2) No
C6A.	Severe complicated measles	(1) Yes (2) No
C6B.	Measles with eye/mouth complications	(1) Yes (2) No
C6C.	Measles	(1) Yes (2) No
C7A.	Acute ear infection	(1) Yes (2) No
C7B.	Chronic ear infection	(1) Yes (2) No
C8A.	Severe complicated malnutrition	(1) Yes (2) No
C8B.	Severe uncomplicated malnutrition	(1) Yes (2) No
C8C.	Moderate acute malnutrition	(1) Yes (2) No
C9A.	Severe anaemia	(1) Yes (2) No
C9B.	Anaemia	(1) Yes (2) No
C10.	Vaccination status **not** up-to-date<br>→ Vaccine(s) needed<br>…………………………………..	(1) Yes (2) No
C11.	Vitamin A status **not** up-to-date	(1) Yes (2) No
C12.	Mebendazole/albendazole supplementation status **not** up-to-date	(1) Yes (2) No
C13.	Other, specify………………	(1) Yes (2) No
C14.	Other, specify………………	(1) Yes (2) No
C15.	Other, specify………………	(1) Yes (2) No

TREATMENT MODULE

Note: If the HEW treats with one dose for a child being referred, record the one dose given (formulation and number of tablets/ml/sachets, etc.) and mark NA for frequency and total days.

T1.	Does the HEW administer or prescribe any treatment or vaccine?	(1) Yes<br>(2) No → ***Skip to T16A***
**Cotrimoxazole**		
T2A.	Does the HEW give cotrimoxazole?	(1) Yes<br>(2) No → ***Skip to T3A***<br>(3) Prescribed only
T2B.	What is the formulation of cotrimoxazole?	(1) Pediatric tablet<br>(2) Adult tablet<br>(3) Syrup → ***Skip to T2D***<br>(8) Other, specify _____________→ ***Skip to T2E***<br>(99) Not specified → ***Skip to T2E***
T2C.	How many cotrimoxazole tablets for each dose?	(1) ½ → ***Skip to T2E***<br>(2) 1 → ***Skip to T2E***<br>(3) 2 → ***Skip to T2E***<br>(4) 3 → ***Skip to T2E***<br>(8) Other, specify ______ → ***Skip to T2E***<br>(99) Not specified → ***Skip to T2E***
T2D.	How many ml of cotrimoxazole syrup for each dose?	(1) 2.5<br>(2) 5<br>(3) 7.5<br>(4) 10<br>(8) Other, specify ______<br>(99) Not specified
T2E.	How many times should cotrimoxazole be given per day?	(1) 1<br>(2) 2<br>(3) 3<br>(8) Other, specify ______<br>(9) NA (referred)<br>(99) Not specified
T2F.	For how many days is cotrimoxazole prescribed?	(1) 3<br>(2) 5<br>(3) 7<br>(8) Other, specify ______<br>(9) NA (referred)<br>(99) Not specified
T2G.	Does the HEW demonstrate how to administer cotrimoxazole?	(1) Yes<br>(2) No
T2H.	Does the HEW ask the caretaker to repeat back how to administer cotrimoxazole?	(1) Yes<br>(2) No
T2I.	Does the HEW give or ask the caretaker to give the first dose of cotrimoxazole before leaving the health post?	(1) Yes<br>(2) No
**ORS**		
T3A.	Does the HEW give ORS?	(1) Yes<br>(2) No → ***Skip to T3G***<br>(3) Prescribed only

T3B.	How many sachets of ORS are given?	(1) 1<br>(2) 2<br>(3) 3<br>(8) Other, specify ______<br>(99) Not specified
T3C.	Does the HEW recommend that the child stay in the health post after the consultation to receive ORS?	(1) Yes<br>(2) No
T3D.	Does the HEW demonstrate how to administer ORS?	(1) Yes<br>(2) No
T3E.	Does the HEW ask the caretaker to repeat back how to administer ORS?	(1) Yes<br>(2) No
T3F.	Does the HEW give or ask the caretaker to give the first dose of ORS before leaving the health post?	(1) Yes<br>(2) No
T3G.	Does the HEW prescribe home-based ORT?	(1) Yes<br>(2) No
**Zinc**		
T4A.	Does the HEW give zinc?	(1) Yes<br>(2) No → ***Skip to T5A***<br>(3) Prescribed only
T4B.	How many zinc tablets for each dose?	(1) 1/2<br>(2) 1<br>(3) 2<br>(8) Other, specify ______<br>(99) Not specified
T4C.	How many times should zinc be given per day?	(1) 1<br>(2) 2<br>(3) 3<br>(8) Other, specify ______<br>(9) NA (referred)<br>(99) Not specified
T4D.	For how many days is zinc prescribed?	(1) 3<br>(2) 5<br>(3) 10<br>(8) Other, specify ______<br>(9) NA (referred)<br>(99) Not specified
T4E.	Does the HEW demonstrate how to administer zinc?	(1) Yes<br>(2) No
T4F.	Does the HEW ask the caretaker to repeat back how to administer zinc?	(1) Yes<br>(2) No
T4G.	Does the HEW give or ask the caretaker to give the first dose of zinc before leaving the health post?	(1) Yes<br>(2) No
**Vitamin A**		
T5A.	Does the HEW give vitamin A?	(1) Yes<br>(2) No → ***Skip to T6A***<br>(3) Prescribed only

T5B.	What is the formulation of vitamin A?	(1) 50,000 IU capsule<br>(2) 100,000 IU capsule<br>(3) 200,000 IU capsule<br>(8) Other, specify ___________________<br>(99) Not specified
T5C.	How many vitamin A capsules does the HEW give?	(1) 1/2<br>(2) 1<br>(3) 2<br>(4) 3<br>(5) 4<br>(8) Other, specify ______<br>(99) Not specified
T5D.	How many doses of vitamin A does the HEW prescribe?	(1) 1<br>(2) 2<br>(3) 3<br>(8) Other, specify ______<br>(99) Not specified
T5E.	Does the HEW give or ask the caretaker to give vitamin A in the health post?	(1) Yes<br>(2) No
T5F.	Does the HEW give vitamin A to be given at home?	(1) Yes<br>(2) No → ***Skip to T6A***
T5G.	Does the HEW demonstrate how to administer vitamin A?	(1) Yes<br>(2) No
T5H.	Does the HEW ask the caretaker to repeat back how to administer vitamin A?	(1) Yes<br>(2) No
**Coartem**		
T6A.	Does the HEW give Coartem?	(1) Yes<br>(2) No → ***Skip to T7A***<br>(3) Prescribed only
T6B.	How many Coartem tablets for each dose?	(1) 1<br>(2) 2<br>(3) 3<br>(4) 4<br>(8) Other, specify ______<br>(99) Not specified
T6C.	How many times should Coartem be given per day?	(1) 1<br>(2) 2<br>(3) 3<br>(8) Other, specify ______<br>(9) NA (referred)<br>(99) Not specified
T6D.	For how many days is Coartem prescribed?	(1) 3<br>(2) 5<br>(3) 7<br>(8) Other, specify ______<br>(9) NA (referred)<br>(99) Not specified
T6E.	Does the HEW demonstrate how to administer Coartem?	(1) Yes<br>(2) No
T6F.	Does the HEW ask the caretaker to repeat back how to administer Coartem?	(1) Yes<br>(2) No

T6G.	Does the HEW give or ask the caretaker to give the first dose of Coartem before leaving the health post?	(1) Yes<br>(2) No
**Chloroquine**		
T7A.	Does the HEW give chloroquine?	(1) Yes<br>(2) No → ***Skip to T8***<br>(3) Prescribed only
T7B.	What is the formulation of chloroquine?	(1) Tablet<br>(2) Syrup → ***Skip to T7D***<br>(8) Other, specify ______________→***Skip to T7E***<br>(99) Not specified →***Skip to T7E***
T7C.	How many chloroquine tablets for each dose?	(1) ¼,¼, ¼ → ***Skip to T7E***<br>(2) ½, ½, ½ → ***Skip to T7E***<br>(3) 1, 1, ½ → ***Skip to T7E***<br>(4) 1,1,1 → ***Skip to T7E***<br>(5) 1½, 1½, 1 → ***Skip to T7E***<br>(6) 2½, 2½, 1 → ***Skip to T7E***<br>(7) 3, 3, 2 → ***Skip to T7E***<br>(7a) 4, 4, 2 → ***Skip to T7E***<br>(8) Other, specify _________ → ***Skip to T7E***<br>(99) Not specified →***Skip to T7E***
T7D.	How many ml of chloroquine syrup for each dose?	(1) 5, 5, 2.5<br>(2) 7.5, 7.5, 5<br>(3) 12.5, 12.5, 7.5<br>(4) 15, 15, 15<br>(5) 20, 20, 15<br>(8) Other, specify ______<br>(99) Not specified
T7E.	How many times should chloroquine be given per day?	(1) 1<br>(2) 2<br>(3) 3<br>(8) Other, specify ______<br>(9) NA (referred)<br>(99) Not specified
T7F.	For how many days is chloroquine prescribed?	(1) 3<br>(2) 5<br>(3) 7<br>(8) Other, specify ______<br>(9) NA (referred)<br>(99) Not specified
T7G.	Does the HEW demonstrate how to administer chloroquine?	(1) Yes<br>(2) No
T7H.	Does the HEW ask the caretaker to repeat back how to administer chloroquine?	(1) Yes<br>(2) No
T7I.	Does the HEW give or ask the caretaker to give the first dose of chloroquine before leaving the health post?	(1) Yes<br>(2) No
**Paracetamol**		
T8.	Does the HEW give paracetamol?	(1) Yes<br>(2) No<br>(3) Prescribed only

Amoxicillin		
T9A.	Does the HEW give amoxicillin?	(1) Yes<br>(2) No → ***Skip to T10A***<br>(3) Prescribed only
T9B.	What is the formulation of amoxicillin?	(1) Tablet<br>(2) Syrup → ***Skip to T9D***<br>(8) Other, specify _______________→ ***Skip to T9F***<br>(99) Not specified → ***Skip to T9F***
T9C.	How many amoxicillin tablets for each dose?	(1) ½ → ***Skip to T9F***<br>(2) 1 → ***Skip to T9F***<br>(3) 2 → ***Skip to T9F***<br>(4) 3 → ***Skip to T9F***<br>(5) 4 → ***Skip to T9F***<br>(8) Other, specify ______ → ***Skip to T9F***<br>(99) Not specified → ***Skip to T9F***
T9D.	What is the strength of the amoxicillin syrup?	(1) 125mg per 5ml<br>(2) 250mg per 5ml<br>(8) Other, specify ______<br>(99) Not specified
T9E.	How many ml of amoxicillin syrup for each dose?	(1) 2.5<br>(2) 5<br>(3) 10<br>(4) 15<br>(5) 20<br>(6) 30<br>(7) 40<br>(8) Other, specify ______<br>(99) Not specified
T9F.	How many times should amoxicillin be given per day?	(1) 1<br>(2) 2<br>(3) 3<br>(8) Other, specify ______<br>(9) NA (referred)<br>(99) Not specified
T9G.	For how many days is amoxicillin prescribed?	(1) 3<br>(2) 5<br>(3) 7<br>(8) Other, specify ______<br>(9) NA (referred)<br>(99) Not specified
T9H.	Does the HEW demonstrate how to administer amoxicillin?	(1) Yes<br>(2) No
T9I.	Does the HEW ask the caretaker to repeat back how to administer amoxicillin?	(1) Yes<br>(2) No
T9J.	Does the HEW give or ask the caretaker to give the first dose of amoxicillin before leaving the health post?	(1) Yes<br>(2) No

**RUTF**		
T10A.	Does the HEW give RUTF (Plumpy Nut or BP 100)?	(1) Yes<br>(2) No → ***Skip to T11***<br>(3) Prescribed only
T10B.	What is the formulation of RUTF?	(1) Plumpy Nut<br>(2) BP 100 → ***Skip to T10D***<br>(8) Other, specify ______________→***Skip to T10E***<br>(99) Not specified →***Skip to T10E***
T10C.	How many sachets of Plumpy Nut per day?	(1) 1¼ → ***Skip to T10E***<br>(2) 1½ → ***Skip to T10E***<br>(3) 2 → ***Skip to T10E***<br>(4) 3 → ***Skip to T10E***<br>(5) 4 → ***Skip to T10E***<br>(6) 5 → ***Skip to T10E***<br>(8) Other, specify ______ → ***Skip to T10E***<br>(9) NA (referred) → ***Skip to T10E***<br>(99) Not specified → ***Skip to T10E***
T10D.	How many bars of BP 100 per day?	(1) 2<br>(2) 2½<br>(3) 4<br>(4) 5<br>(5) 7<br>(6) 9<br>(8) Other, specify ______<br>(9) NA (referred)<br>(99) Not specified
T10E.	For how many days is RUTF prescribed?	______<br>(99) NA (referred)<br>(999) Not specified
T10F.	Does the HEW give or ask the caretaker to give RUTF before leaving the health post?	(1) Yes<br>(2) No
**Folic Acid**		
T11.	Does the HEW give folic acid?	(1) Yes<br>(2) No<br>(3) Prescribed only
**Mebendazole/Albendazole**		
T12A.	Does the HEW give mebendazole or albendazole?	(1) Yes<br>(2) No → ***Skip to T13***<br>(3) Prescribed only
T12B.	What is the formulation of mebendazole/ albendazole?	(1) Mebendazole 100 mg tablet<br>(2) Mebendazole 500 mg tablet<br>(3) Albendazole 400 mg tablet<br>(8) Other, specify ___________________<br>(99) Not specified

T12C.	How many tablets of mebendazole/albendazole does the HEW give?	(1) 1<br>(2) 2<br>(3) 3<br>(4) 4<br>(5) 5<br>(8) Other, specify ______<br>(99) Not specified
**Tetracycline**		
T13.	Does the HEW give tetracycline ointment?	(1) Yes<br>(2) No<br>(3) Prescribed only
**Other Treatments**		
T14.	Does the HEW give other treatments?<br>Specify: …………………………<br>…………………………<br>…………………………	(1) Yes<br>(2) No<br>(3) Prescribed only
**Vaccines**		
T15.	Does the HEW give vaccines?<br>Specify: ………………………<br>………………………<br>………………………	(1) Yes<br>(2) No<br>(3) Prescribed only
**Referral**		
T16A.	Does the HEW refer the child to a health facility?	(1) Yes<br>(2) No → ***Skip to T17A***
T16B.	Does the caretaker accept referral for the child?	(1) Yes<br>(2) No, specify reason ………………………………
T16C.	What was the reason for referral?	(1) Severe illness<br>(2) Drug stock-out<br>(8) Other, specify reason ……………………………
T16D.	Does the HEW explain the need for referral?	(1) Yes<br>(2) No
T16E.	Does the HEW write a referral note?	(1) Yes<br>(2) No
T16F.	Does the HEW arrange transportation?	(1) Yes<br>(2) No
**Advising on Home Care**		
T17A.	Does the HEW advise on home care?	(1) Yes<br>(2) No → ***Skip to T18A***
T17B.	Does the HEW advise to go to health facility/return if the child cannot drink or breastfeed?	(1) Yes<br>(2) No
T17C.	Does the HEW advise to go to health facility/return if child becomes sicker?	(1) Yes<br>(2) No
T17D.	Does the HEW advise caregiver to increase fluids?	(1) Yes<br>(2) No
T17E.	Does the HEW advise caregiver to continue feeding?	(1) Yes<br>(2) No
T17F.	Does the HEW advise to continue breastfeeding and/or breastfeed more frequently?	(1) Yes<br>(2) No

T17G.	Does the HEW advise on when to return for follow-up?	(1) Yes<br>(2) No
**Job Aids**		
T18A.	Does the HEW use the iCCM chart booklet at any time during the encounter with the child?	(1) Yes (2) No
T18B.	Does the HEW use the iCCM registration book at any time during the encounter with the child?	(1) Yes (2) No

Note: After the consultation, if the treatment is not clear, ask the health worker what treatments were given or prescribed to the patient. Ask "Any other treatments?" until the health worker has stated all treatments given/prescribed. Do not ask for each specific treatment.

Time of end of observation: …………..

Calculate total time of observation: ……… minutes

END OF OBSERVATION

Form 2. Caretaker Exit Interview – child (2–59 months)

Fill out one Exit Interview per sick child observed

Unique child ID: ☐☐☐☐ **Unique HEW ID**: ☐☐☐ Data collector name: ………………

Zone: …………………. Woreda: …………………. Kebele: …………………. Health post: ………………….

HEW number: …………………. HEW name: ………………….

Child name: …………………. Child number: ……….. Caretaker number: ………………….

EX1. Did the HEW give you or prescribe any medicines for <CHILD> today?
HEF hara'a daa'imaaf dawaa/goricha keenitee ykn ajajjeettii?
→ *Note: 'Medicines' excludes ORS. See EX7 for ORS*
→ *Note: Medicines prescribed include only those received by or prescribed to the caretaker for home care.*

(1) Yes
(2) No → ***Skip to EX7A***
(3) Referred → ***Skip to RE1 (Re-examination Form)***

EX2A. What was the FIRST medicine prescribed or given?
→ *Copy the information from the caretaker's medication or prescription:*

(1) Cotrimoxazole
(2) Zinc
(3) Vitamin A
(4) Coartem
(5) Chloroquine
(6) Paracetamol
(7) Amoxicillin
(8) Plumpy Nut
(9) BP 100
(10) Folic acid
(11) Mebendazole
(12) Albendazole
(88) Other, specify ……………………………………

EX2B. What was the formulation?
→ *Copy the information from the caretaker's medication or prescription:*

(1) Tablet
(2) Syrup
(3) Capsule
(4) Sachet
(5) Bar
(8) Other, specify ……………………………………

EX2C. How much will you give <CHILD> each time?
Daa'imaaf si'a tokkotti hagam kenniitaa?

……………………………………

(999) Don't know

EX2D. How many times will you give it to <CHILD> each day?
Guyyaatti si'a meeqa kenniitaa?

(1) 1
(2) 2
(3) 3
(4) 4
(5) 5
(8) Other, specify ………
(9) Don't know

EX2E. How many days will you give the medicine to <CHILD>?
Daa'immaf Dawaa/goricha guyyaa meeqaaf kennitta?

(1) 1
(2) 2
(3) 3
(4) 4
(5) 5
(6) 6
(7) 7
(8) Other, specify ………
(9) Don't know

EX3A. Was a SECOND medicine prescribed or given?
Dawaan/gorichi lammataa kennamee ykn ajajameeraa?

(1) Yes
(2) No → ***Skip to EX7A***

EX3B. What was the SECOND medicine prescribed or given?
→ *Copy the information from the caretaker's medication or prescription:*

(1) Cotrimoxazole
(2) Zinc
(3) Vitamin A
(4) Coartem
(5) Chloroquine
(6) Paracetamol
(7) Amoxicillin
(8) Plumpy Nut
(9) BP 100
(10) Folic acid
(11) Mebendazole
(12) Albendazole
(88) Other, specify ……………………………………

EX3C. What was the formulation?
→ *Copy the information from the caretaker's medication or prescription:*

(1) Tablet
(2) Syrup
(3) Capsule
(4) Sachet
(5) Bar
(8) Other, specify ……………………………………

EX3D. How much will you give <CHILD> each time?

Daa'imaaf si'a tokkotti hagam kenniitaa?

………………………………………

(999) Don't know

EX3E. How many times will you give it to <CHILD> each day?

Guyyaatti si'a meeqa kenniitaa?

(1) 1
(2) 2
(3) 3
(4) 4
(5) 5
(8) Other, specify ………
(9) Don't know

EX3F. How many days will you give the medicine to <CHILD>?

Daa'immaf Dawaa/goricha guyyaa meeqaaf kennitta?

(1) 1
(2) 2
(3) 3
(4) 4
(5) 5
(6) 6
(7) 7
(8) Other, specify ………
(9) Don't know

EX4A. Was a THIRD medicine prescribed or given?

Dawaan/gorichi sadafaa kennamee ykn ajajameeraa?

(1) Yes
(2) No → ***Skip to EX7A***

EX4B. What was the THIRD medicine prescribed or given?

→ *Copy the information from the caretaker's medication or prescription:*

(1) Cotrimoxazole
(2) Zinc
(3) Vitamin A
(4) Coartem
(5) Chloroquine
(6) Paracetamol
(7) Amoxicillin
(8) Plumpy Nut
(9) BP 100
(10) Folic acid
(11) Mebendazole
(12) Albendazole
(88) Other, specify …………………………………….

EX4C. What was the formulation?

→ *Copy the information from the caretaker's medication or prescription:*

(1) Tablet
(2) Syrup
(3) Capsule
(4) Sachet
(5) Bar
(8) Other, specify ……………………………………

EX4D. How much will you give <CHILD> each time?
Daa'imaaf si'a tokkotti hagam kenniitaa?

………………………………………

(999) Don't know

EX4E. How many times will you give it to <CHILD> each day?
Guyyaatti si'a meeqa kenniitaa?

(1) 1
(2) 2
(3) 3
(4) 4
(5) 5
(8) Other, specify ………
(9) Don't know

EX4F. How many days will you give the medicine to <CHILD>?
Daa'immaf Dawaa/goricha guyyaa meeqaaf kennitta?

(1) 1
(2) 2
(3) 3
(4) 4
(5) 5
(6) 6
(7) 7
(8) Other, specify ………
(9) Don't know

EX5A. Was a FOURTH medicine prescribed or given?
Dawaan/gorichi afuraffaa ajajameeraa ykn kennameeraa?

(1) Yes
(2) No → ***Skip to EX7A***

EX5B. What was the FOURTH medicine prescribed or given?
→ *Copy the information from the caretaker's medication or prescription:*

(1) Cotrimoxazole
(2) Zinc
(3) Vitamin A
(4) Coartem
(5) Chloroquine
(6) Paracetamol
(7) Amoxicillin
(8) Plumpy Nut
(9) BP 100
(10) Folic acid
(11) Mebendazole
(12) Albendazole
(88) Other, specify ……………………………………

EX5C. What was the formulation?
→ *Copy the information from the caretaker's medication or prescription:*

(1) Tablet
(2) Syrup
(3) Capsule
(4) Sachet
(5) Bar
(8) Other, specify ……………………………………

EX5D. How much will you give <CHILD> each time?
Daa'imaaf si'a tokkotti hagam kenniitaa?

………………………………………

(999) Don't know

EX5E. How many times will you give it to <CHILD> each day?
Guyyaatti si'a meeqa kenniitaa?

(1) 1
(2) 2
(3) 3
(4) 4
(5) 5
(8) Other, specify ………
(9) Don't know

EX5F. How many days will you give the medicine to <CHILD>?
Daa'immaf Dawaa/goricha guyyaa meeqaaf kennitta?

(1) 1
(2) 2
(3) 3
(4) 4
(5) 5
(6) 6
(7) 7
(8) Other, specify ………
(9) Don't know

EX6A. Was a FIFTH medicine prescribed or given?

Dawaan/gorichi Shanafaa kennameeraa ykn ajajameeraa?

(1) Yes

(2) No → ***Skip to EX7A***

EX6B. What was the FIFTH medicine prescribed or given?

→ *Copy the information from the caretaker's medication or prescription:*

(1) Cotrimoxazole
(2) Zinc
(3) Vitamin A
(4) Coartem
(5) Chloroquine
(6) Paracetamol
(7) Amoxicillin
(8) Plumpy Nut
(9) BP 100
(10)Folic acid
(11)Mebendazole
(12)Albendazole
(88)Other, specify ……………………………………

EX6C. What was the formulation?

→ *Copy the information from the caretaker's medication or prescription:*

(1) Tablet
(2) Syrup
(3) Capsule
(4) Sachet
(5) Bar
(8) Other, specify ……………………………………

EX6D. How much will you give <CHILD> each time?

Daa'imaaf si'a tokkotti hagam kenniitaa?

………………………………………

(999) Don't know

EX6E. How many times will you give it to <CHILD> each day?

Guyyaatti si'a meeqa kenniitaa?

(1) 1
(2) 2
(3) 3
(4) 4
(5) 5
(8) Other, specify ………
(9) Don't know

EX6F. How many days will you give the medicine to <CHILD>?
Daa'immaf Dawaa/goricha guyyaa meeqaaf kennitta?

(1) 1
(2) 2
(3) 3
(4) 4
(5) 5
(6) 6
(7) 7
(8) Other, specify ………
(9) Don't know

EX7A. Was ORS prescribed or given?

(1) Yes
(2) No → ***Skip to EX8***

EX7B. How much water will you mix with one ORS packet?
Paakeetii ORS tokko bishaan hagamiin bulbulta?

…………… liters

(999) Don't know

EX8. Did the HEW give you a specific day when to come back to the health post?
HEF guyyaa gara kellaa fayyaa itti deebitanii dhuftan isiniitti himteettii?

(1) Yes → In how many days? ………. days
(2) No
(9) Don't know

END OF EXIT INTERVIEW

Thank the caretaker for answering your questions and ask if he/she has any questions. Be sure that the caretaker knows how to prepare ORS for a child with diarrhoea, when to return for vaccination, how to give the prescribed medications, and when to return if the child becomes worse at home.

Form 3. Re-examination Form – child (2 – 59 months)

Fill out one Re-examination Form per sick child observed

Unique child ID: ☐☐☐☐ **Unique HEW ID**: ☐☐☐ Data collector name: ………………

Zone: …………………. Woreda: …………………. Kebele: …………………. Health post: ………………….

HEW number: …………………. HEW name: ………………….

Caretaker number: ………………….

Child name: …………………. Child number: ……….. Child's age (completed months): …………..

RE1. Ask what the child's problems are.
Circle all signs mentioned.

A.	Fast/difficult breathing	(1) mentioned	(2) not mentioned
B.	Cough	(1) mentioned	(2) not mentioned
C.	Pneumonia	(1) mentioned	(2) not mentioned
D.	Diarrhoea	(1) mentioned	(2) not mentioned
E.	Fever	(1) mentioned	(2) not mentioned
F.	Malaria	(1) mentioned	(2) not mentioned
G.	Convulsions	(1) mentioned	(2) not mentioned
H.	Difficulty drinking or breastfeeding	(1) mentioned	(2) not mentioned
I.	Vomiting	(1) mentioned	(2) not mentioned
J.	Ear problem	(1) mentioned	(2) not mentioned
K.	Other problem, specify:	(1) mentioned	(2) not mentioned
L.	Other problem, specify:	(1) mentioned	(2) not mentioned
M.	Other problem, specify:	(1) mentioned	(2) not mentioned

**Assess**			**Classify**	
**Take the child's temperature and weight**<br>RE2A. Temperature: ……...<br>RE2B. Weight: ……...				
**Check for general danger signs**			RE3E.	**General danger sign present?**<br>(1) Yes<br>(2) No<br><br>*Note: Remember to use danger sign when selecting classifications*
RE3A.	Is the child **unable** to drink or breastfeed?<br>*Daa'imni harma hodhuu ykn waa dhuguu hindanda'uu?*	(1) Yes<br>(2) No		
RE3B.	Does the child vomit EVERYTHING?<br>Daa'imn*i waa hunda hooqqisaa?*	(1) Yes<br>(2) No		
RE3C.	Has the child had convulsions?<br>*Daa'imni ni gagaba/ni urgufamaa?*	(1) Yes<br>(2) No		

RE3D.	Is the child lethargic or unconscious?<br>*Daa'imni of wallaalaa?*	(1) Yes<br>(2) No		
**Check for pneumonia**			RE4F.	**Pneumonia classification:**<br>(1) Severe pneumonia/very severe disease<br>(2) Pneumonia<br>(3) No pneumonia: cough or cold
RE4A.	Does the child have cough or difficult breathing?<br>*Daa'imni qufaa ykn rakkoo argansuu afuuraa qabaa?*	(1) Yes<br>(2) No →***Skip to RE5A***		
RE4B.	How long has the child had cough or difficult breathing?<br>*Qufaan ykn rakkoon argansuu afuuraa hagam irrati ture?*	.... days		
RE4C.	How many breaths does the child have in 1 minute?	…........ bpm		
RE4D.	Does the child have chest indrawing?	(1) Yes<br>(2) No		
RE4E.	Does the child have stridor?	(1) Yes<br>(2) No		
**Check for diarrhoea and dehydration**			RE5J.	**Diarrhoea classification:**<br>(1) Diarrhoea, severe dehydration<br>(2) Diarrhoea, some dehydration<br>(3) Diarrhoea, no dehydration<br>(4) Severe persistent diarrhoea<br>(5) Persistent diarrhoea<br>(6) Dysentery
RE5A.	Does the child have diarrhoea?<br>*Daa'imni garaa kaasaa qabaa?*	(1) Yes<br>(2) No →***Skip to RE6A***		
RE5B.	How long has the child had diarrhoea?<br>*Garaa kaasaan daa'ima irra yeroo hagam ture?*	.... days		
RE5C.	Is there blood in the stool?<br>*Boolii isaa keessa dhiigni nijirra?*	(1) Yes<br>(2) No		
RE5D.	Is the child restless or irritable?<br>*Daa'imni ni aaraa ykn jijiraa amalaa niqabaa?*	(1) Yes<br>(2) No		
RE5E.	Does the child have sunken eyes?	(1) Yes<br>(2) No		
RE5F.	Is the child **not** able to drink or drinking poorly?<br>*Daa'imni dhuguu hindanda'uu moo ykn xiino dhugaa?*	(1) Yes<br>(2) No		
RE5G.	Is the child drinking eagerly, thirsty?<br>*(Offer the child water to drink)*	(1) Yes<br>(2) No		
RE5H.	Does the abdomen skin pinch go back slowly (less than 2 seconds)?	(1) Yes<br>(2) No		
RE5I.	Does the abdomen skin pinch go back very slowly (longer than 2 seconds)?	(1) Yes<br>(2) No		
**Check for malaria**			RE6J.	**Malaria classification:**<br>(1) Very severe febrile disease<br>(2) Malaria<br>(3) Fever: malaria unlikely<br>(4) Fever: no malaria
RE6A.	Does the child have fever or history of fever (last 48 hrs)?<br>*Daa'imni gubaa qaamaa qabaa ykn sa'atii 48 darban keessatti qabaa?*	(1) Yes<br>(2) No →***Skip to RE8A***		
RE6B.	How long has the child had fever?<br>*Gubaan qaama hagam irra ture?*	.... days →***If less than 8 days, skip to RE6D***		

RE6C.	Was the fever present every day?<br>*Gubaan qaama guyyaa guyyaan isa mudataa?*	(1) Yes<br>(2) No		
RE6D.	Was an RDT performed for the child by the HEW?	(1) Yes →***Skip to RE6F***<br>(2) No<br>(3) Yes, but incorrectly		
RE6E.	Was an RDT performed for the child by the re-examiner?<br>*Note: RDT should be performed in low or high malaria area or if child has traveled to a malarious area in the 2 weeks before the illness began.*	(1) Yes<br>(2) No →***Skip to RE6G***		
RE6F.	What is the child's RDT result?	(1) Positive falciparum<br>(2) Positive vivax<br>(3) Positive falc. & vivax<br>(4) Negative		
RE6G.	Does the child have a stiff neck?	(1) Yes<br>(2) No		
RE6H.	If child is **less than 1 year**, does the child have bulged fontanel?	(1) Yes<br>(2) No<br>(9) NA		
RE6I.	Does the child have runny nose?	(1) Yes<br>(2) No		
**Check for measles**			RE7E.	**Measles classification:**<br>(1) Severe complicated measles<br>(2) Measles with eye or mouth complications<br>(3) Measles
RE7A.	Has the child had measles in the last 3 months or does the child have signs of measles (generalized rash and one of these: cough, runny nose, red eyes)?<br>*Baatii sadan darban keessatti daa'imni gifirri/shiftoon ykn mallattoon gifiraa irratti mul'ateeraa?*	(1) Yes<br>(2) No →***Skip to RE8A***		
RE7B.	Does the child have mouth ulcers?	(1) Yes<br>(2) No		
RE7C.	Does the child have pus draining from the eye?	(1) Yes<br>(2) No		
RE7D.	Does the child have clouding of the cornea?	(1) Yes<br>(2) No		
**Check for ear infection**			RE8E.	**Ear infection classification:**<br>(1) Acute ear infection<br>(2) Chronic ear infection<br>(3) No ear infection
RE8A.	Does the child have an ear problem?<br>*Daa'imni rakkoo gurraa qabaa?*	(1) Yes<br>(2) No →***Skip to RE9A***		
RE8B.	Is there ear pain?<br>*Gurra ni dhukkubaa?*	(1) Yes<br>(2) No		
RE8C.	Is there ear discharge?<br>*Gurri ni malaa'aa?*	(1) Yes<br>(2) No →***Skip to RE8E***		
RE8D.	How long has the child had ear discharge?<br>*Gurri mala'uu erga jalqabee hagam ta'eera?*	.... days		

Check for malnutrition

RE9A.	Does child have pitting edema of both feet?	(1) Yes<br>(2) No
RE9B.	If **younger than 6 months**, does child have visible severe wasting?	(1) Yes<br>(2) No<br>(9) NA
RE9C.	If **6 months or older**, what is the child's MUAC measurement?	(1) <11 cm<br>(2) 11-<12 cm<br>(3) ≥12 cm<br>(9) NA
RE9D.	If **6 months or older** AND MUAC < 11cm OR bilateral oedema, did the child pass an appetite test?	(1) Yes<br>(2) No<br>(9) NA
RE9E.	If **6 months or older**, does the child have any complicating condition? (pneumonia, watery diarrhoea, dysentery, fever/low temperature)	(1) Yes<br>(2) No<br>(9) NA

RE9F.	**Malnutrition classification:**<br>(1) Severe complicated malnutrition<br>(2) Severe uncomplicated malnutrition<br>(3) Moderate acute malnutrition<br>(4) No acute malnutrition

Check for anaemia

RE10A.	Does the child have severe palmar pallor?	(1) Yes<br>(2) No
RE10B.	Does the child have moderate palmar pallor?	(1) Yes<br>(2) No

RE10C.	**Anaemia classification:**<br>(1) Severe anaemia<br>(2) Anaemia<br>(3) No anaemia

Check immunization status

Review the child's immunization card. If the card is not available, probe the caretaker using the immunization questions provided to obtain as much information as possible regarding the child's vaccines.

RE11A.	If the child is **under 24 months**, has the child received all appropriate vaccines?	(1) Yes→***Skip to RE11C***<br>(2) No<br>(9) NA→***Skip to RE12A***
RE11B.	*Circle needed vaccines*<br><br>**Birth** BCG OPV-0<br>**6 weeks** Penta-1 Pneumoc.-1 OPV-1<br>**10 weeks** Penta-2 Pneumoc.-2 OPV-2<br>**14 weeks** Penta-3 Pneumoc.-3 OPV-3<br>**9 months** Measles<br><br>*Note: Keep an interval of 4 weeks between Penta and OPV doses. Do not recommend OPV-0 if the child is 14 days old or more.*	

RE11C.	**Immunization classification:**<br>(1) Vaccination status up-to-date<br>(2) Vaccination status **not** up-to-date<br>(3) Completed<br>(4) Not started

Check vitamin A supplementation status

RE12A.	If **6 months or older**, has the child received a vitamin A supplementation in the previous 6 months?<br>*Yoo daa'imni Ji'a 6 ykn sanaa ol ta'e: ji'ottan jahan darban keessatti daa'imni qooricha Vitaamina A fudhateeraa?*	(1) Yes<br>(2) No<br>(9) NA →***Skip to RE13A***

RE12B.	**Vitamin A classification:**<br>(1) Vitamin A supplementation status up-to-date<br>(2) Vitamin A supplementation status **not** up-to-date

<table>
<tr><td colspan="3">Check mebendazole/albendazole status</td><td rowspan="2">RE13B.</td><td rowspan="2">Mebendazole/ albendazole classification:
(1) Mebendazole/ albendazole status up-to-date
(2) Mebendazole/ albendazole status not up-to-date</td></tr>
<tr><td>RE13A.</td><td>If <u>24 months or older</u>, has the child received mebendazole/albendazole in the previous 6 months?
Yoo daa’imni Ji’a 24 ykn sanaa ol ta’e; baatii 6 haan darban keessatti mebendazolii/albendazolii yokin qoricha rammoo garaa fudhateeraa?</td><td>(1) Yes
(2) No
(9) NA →End re-examination</td></tr>
</table>

Once the re-examination is finished, ask the child and caretaker to wait. Write down the treatments needed for the child according to the iCCM guidelines. Then check the treatment given or prescribed by the HEW. If there is any discrepancy between the HEW’s treatments and the re-examiner’s treatments, discuss this discrepancy with the HEW and ensure that the child receives the correct treatments.

END OF RE-EXAMINATION FORM

Form 4. Equipment, Supplies & Support Checklist

Fill out one Equipment, Supplies & Support Checklist per health post

Zone: ………………… Woreda: ………………… Kebele: ………………… Health post: …………………

Data collector: ………………

DRUGS & SUPPLIES MODULE

*Ask the HEW to show you around where she works. Check the drug stocks and other supplies. Answer the following questions based on what you **see**.*

D1. Does the health post have the following (unexpired) drugs/diagnostics available the day of visit?

A.	Cotrimoxazole	(1) Yes<br>(2) No	Amount in stock: ……….. tablets<br>Amount in stock: ……….. bottles syrup
B.	ORS	(1) Yes<br>(2) No	Amount in stock: ……….. sachets
C.	Zinc	(1) Yes<br>(2) No	Amount in stock: ……….. tablets
D.	Vitamin A	(1) Yes<br>(2) No	Amount in stock: ……….. capsules
E.	Coartem	(1) Yes<br>(2) No	Amount in stock: ……….. tablets
F.	Chloroquine	(1) Yes<br>(2) No	Amount in stock: ……….. tablets<br>Amount in stock: ……….. bottles syrup
G.	Paracetamol	(1) Yes<br>(2) No	Amount in stock: ……….. tablets<br>Amount in stock: ……….. bottles syrup
H.	Amoxicillin	(1) Yes<br>(2) No	Amount in stock: ……….. tablets<br>Amount in stock: ……….. bottles syrup
I.	Plumpy Nut	(1) Yes<br>(2) No	Amount in stock: ……….. sachets
J.	BP 100	(1) Yes<br>(2) No	Amount in stock: ……….. bars
K.	Folic Acid	(1) Yes<br>(2) No	Amount in stock: ……….. tablets
L.	Mebendazole/albendazole	(1) Yes<br>(2) No	Amount in stock: ……….. tablets
M.	Tetracycline ointment	(1) Yes<br>(2) No	Amount in stock: ……….. tubes
N.	Vitamin K	(1) Yes<br>(2) No	Amount in stock: ……….. ampules

O.	RDT	(1) Yes<br>(2) No	Amount in stock: ……….. tests

D2. Does the health post have the following <u>EXPIRED</u> drugs/diagnostics on the day of visit?

A.	Cotrimoxazole	(1) Yes<br>(2) No	Amount expired: ……….. tablets<br>Amount expired: ……….. bottles syrup
B.	ORS	(1) Yes<br>(2) No	Amount expired: ……….. sachets
C.	Zinc	(1) Yes<br>(2) No	Amount expired: ……….. tablets
D.	Vitamin A	(1) Yes<br>(2) No	Amount expired: ……….. capsules
E.	Coartem	(1) Yes<br>(2) No	Amount expired: ……….. tablets
F.	Chloroquine	(1) Yes<br>(2) No	Amount expired: ……….. tablets<br>Amount expired: ……….. bottles syrup
G.	Paracetamol	(1) Yes<br>(2) No	Amount expired: ……….. tablets<br>Amount expired: ……….. bottles syrup
H.	Amoxicillin	(1) Yes<br>(2) No	Amount expired: ……….. tablets<br>Amount expired: ……….. bottles syrup
I.	Plumpy Nut	(1) Yes<br>(2) No	Amount expired: ……….. sachets
J.	BP 100	(1) Yes<br>(2) No	Amount expired: ……….. bars
K.	Folic Acid	(1) Yes<br>(2) No	Amount expired: ……….. tablets
L.	Mebendazole/albendazole	(1) Yes<br>(2) No	Amount expired: ……….. tablets
M.	Tetracycline ointment	(1) Yes<br>(2) No	Amount in stock: ……….. tubes
N.	Vitamin K	(1) Yes<br>(2) No	Amount in stock: ……….. ampules
O.	RDT	(1) Yes<br>(2) No	Amount expired: ……….. tests

D3. Does the health post have the following equipment and supplies?

A. Functional timer (that can accurately count a minute) (1) Yes (2) No
B. Functional thermometer (1) Yes (2) No
C. Functional scale (1) Yes (2) No
D. 1 liter measuring container (1) Yes (2) No
E. Clean water in a container (1) Yes (2) No
F. Cup for ORS (1) Yes (2) No
G. Spoon for ORS (1) Yes (2) No
H. Tray for ORT corner (1) Yes (2) No
I. Cloth for ORT corner (1) Yes (2) No
J. MUAC tape (1) Yes (2) No
K. Ambu bag (1) Yes (2) No

D4. Does the health post have the following job aids available the day of visit?

A. iCCM chart booklet (1) Yes (2) No
B. iCCM registration book (both age groups) (1) Yes (2) No
C. Family health card (1) Yes (2) No
D. OTP card (1) Yes (2) No

Ask the following questions to the HEW. Use any documentation available (e.g. drug stock cards) to help determine this information.

D5. Have you experienced a stock-out in the last three months of any of the following? If yes, what was the longest number of consecutive days without that item?

A.	Cotrimoxazole	(1) Yes (2) No (3) never rcvd.	Duration of stock-out ……… days
B.	ORS	(1) Yes (2) No (3) never rcvd.	Duration of stock-out ……… days
C.	Zinc	(1) Yes (2) No (3) never rcvd.	Duration of stock-out ……… days
D.	Vitamin A	(1) Yes (2) No (3) never rcvd.	Duration of stock-out ……… days
E.	Coartem	(1) Yes (2) No (3) never rcvd.	Duration of stock-out ……… days
F.	Chloroquine	(1) Yes (2) No (3) never rcvd.	Duration of stock-out ……… days
G.	Paracetamol	(1) Yes (2) No (3) never rcvd.	Duration of stock-out ……… days
H.	Amoxicillin	(1) Yes (2) No (3) never rcvd.	Duration of stock-out ……… days
I.	RUTF	(1) Yes (2) No (3) never rcvd.	Duration of stock-out ……… days
J.	Folic acid	(1) Yes (2) No (3) never rcvd.	Duration of stock-out ……… days
K.	Mebendazole/albendazole	(1) Yes (2) No (3) never rcvd.	Duration of stock-out ……… days
L.	Tetracycline ointment	(1) Yes (2) No (3) never rcvd.	Duration of stock-out ……… days
M.	Vitamin K	(1) Yes (2) No (3) never rcvd.	Duration of stock-out ……… days
N.	RDT	(1) Yes (2) No (3) never rcvd.	Duration of stock-out ……… days

SERVICES & SUPPORT MODULE

S1. How many hours was the health post open last week (Monday-Friday)?
Torban darbe kellaan fayyaa sa'atii meeqaaf banaa ture (Wiixata – Jimaataa)?

…………… hours (999) Don't know

S2. How many volunteer community health workers (VCHWs) are there in the health post catchment area?
Keellaa fayyaa kana jala hojjettoota fedhii hawaasaa meeqatu jira?

…………… VCHWs (999) Don't know

S3. Which of the following community education/mobilization activities focused on iCCM were carried out by HEWs or VCHWs in the last month?
Read list to HEW

A.	Health education during Idir	(1) Yes (2) No (999) DK	Number reported ………	People reported ……….	Number documented ………	People documented ……….
B.	Community coversation enhancement	(1) Yes (2) No (999) DK	Number reported ………	People reported ……….	Number documented ………	People documented ……….
C.	Community leader meeting	(1) Yes (2) No (999) DK	Number reported ………	People reported ……….	Number documented ………	People documented ……….
D.	Outreach community mobilization	(1) Yes (2) No (999) DK	Number reported ………	People reported ……….	Number documented ………	People documented ……….
E.	Growth monitoring and promotion	(1) Yes (2) No (999) DK	Number reported ………	People reported ……….	Number documented ………	People documented ……….
F.	Other, specify …………………………………	(1) Yes (2) No (999) DK	Number reported ………	People reported ……….	Number documented ………	People documented ……….
G.	Other, specify …………………………………	(1) Yes (2) No (999) DK	Number reported ………	People reported ……….	Number documented ………	People documented ……….

S4. How many times during the last <u>three months</u> did you receive a supervisory visit that included supervision of iCCM activities?
Daawwannaa (supparviziniii) sochii ICCM dabalatee, Ji'a sadan darban keessatti si'a meeqa siif godhame?
Probe the HEW to ensure that the HEW understands that this refers to supervision visits where the supervisor comes to talk about iCCM and the HEWs' management of sick children.

…………… times → ***<u>if 0 times, skip to S7</u>***

(99) NA (HEWs not present in HP majority of last 3 months) → ***skip to S7***

(999) Don't know → ***skip to S7***

S5. At these supervision visits in the last three months that included supervision of iCCM activities, who visited you?
Baatiiwwan sadan darban keessatti sochii ICCM dabalatee, qaamni isin daawwate eenyufaadha.
Circle all responses given, do not read list

A. Supervisor from the supporting health center

B. Woreda supervisor

C. NGO supervisor

D. Other, specify ……………………………………..

S6. At any of these visits, did the supervisor(s) do the following?
Yeroo daawwannaa kana keessatti supparvaayizarootni waan kanaa gadii hojjetaniiru?
Read list to HEW

A.	Observe you managing a sick child<br>*Yeroo ati daa'ima dhibamaa wal'aantu si ilaalaniiru?*	(1) Yes	(2) No	(9) Don't know
B.	Use a supervision checklist<br>*Cheekliistii supparviizyirii fayyadamaniiru*	(1) Yes	(2) No	(9) Don't know
C.	Review the iCCM registration book<br>*Galmee ICCM ni ilaaluu*	(1) Yes	(2) No	(9) Don't know
D.	Provide verbal feedback<br>*Dub deebii Afaanii sii kennaniiruu*	(1) Yes	(2) No	(9) Don't know
E.	Provide written feedback<br>*Dub deebii barreeffamaan sii kennaniiruu*	(1) Yes	(2) No	(9) Don't know

S7. Where do you refer children with danger signs?
Daa'imman mallattoo hamaa irratti agarte eessatti ergita (riifarii) goota?

(1) Health center, specify name: ………………………

(2) Hospital, specify name: ……………………………

(8) Other, specify: ………………………………………

S8. How long does it usually take for a patient to get to the nearest referral location (using the most common means of transport)?
Bakka dhaabbata fayyaa ergite (riifarii goote) ga'uuf dhibamaa hagam itti fudhata.(hagam fagaata?)

…………hours ………mins (999) Don't know

HEALTH POST RECORDS MODULE

Ask if you can look at the health post iCCM registers. Use the ***iCCM registration books*** *to answer the questions below. Use the last completed month. Do not rely on the HEWs' monthly report. Go through the cases in the register to add up the numbers.*

R1. Is there an iCCM registration book available in the health post?

(1) Yes

(2) No → ***Skip to R11***

R2. What is the number of initial consultations for children 0 – 59 months recorded in the register for the previous month by sex?

A. Female …………

B. Male …………

C. Unspecified …………

R3. How many of these initial consultations were for children between the ages of 0 days – 2 months in the previous month?

Initial consultations for children 0 days – 2 months: …………

R4. How many sick children (0 – 59 months) were referred in the previous month?

Children referred: …………

R5. In the previous month, how many children (0 – 59 months) were classified as having:

A. Pneumonia? …………

B. Diarrhoea? …………

C. Malaria? …………

D. Ear infection? …………

E. Acute malnutrition? …………

F. Anaemia? …………

R6. In the previous month, how many children (0 – 59 months) received:

A. Antibiotic for pneumonia? …………

B. ORS for diarrhoea? …………

C. Zinc for diarrhoea? …………

D. ACT for malaria? …………

E. Chloroquine for malaria? …………

F. RUTF for acute malnutrition? …………

G. Antibiotic for acute malnutrition? …………

R7. Are there any sick children 0 days – 2 months registered in the iCCM register?

(1) Yes

(2) No → ***Skip to R9***

R8. Record the following information for the past 10 cases of sick children with iCCM illness <u>0 days – 2 months</u> with an iCCM illness.

Include patients receiving initial consultation and having at least one iCCM-related symptom.

	Date Seen	Age (weeks) & Sex	Weight & Temperature	Signs & Symptoms (Select from list of complaints)	Disease Classification (Select from list of classifications)	Treatment Given (drug, dose, schedule, duration)	Referred?	Outcome Registered?
R8A.	…../…../…..	Age:<br>M F	Weight:<br>Temp:				Y N	Y N
R8B.	…../…../…..	Age:<br>M F	Weight:<br>Temp:				Y N	Y N
R8C.	…../…../…..	Age:<br>M F	Weight:<br>Temp:				Y N	Y N
R8D.	…../…../…..	Age:<br>M F	Weight:<br>Temp:				Y N	Y N
R8E.	…../…../…..	Age:<br>M F	Weight:<br>Temp:				Y N	Y N

R8F.	…../…../…..	Age:<br>M F	Weight:<br>Temp:				Y N	Y N
R8G.	…../…../…..	Age:<br>M F	Weight:<br>Temp:				Y N	Y N
R8H.	…../…../…..	Age:<br>M F	Weight:<br>Temp:				Y N	Y N
R8I.	…../…../…..	Age:<br>M F	Weight:<br>Temp:				Y N	Y N
R8J.	…../…../…..	Age:<br>M F	Weight:<br>Temp:				Y N	Y N

R9. Are there any sick children 2 – 59 months registered in the iCCM register?

(1) Yes

(2) No → ***Skip to R11***

R10. Record the following information for the past 10 cases of sick children 2 – 59 months with an iCCM illness.

Include patients receiving initial consultation and having at least one iCCM-related symptom.

	Date Seen	Age (months) & Sex	Weight & Temperature	Signs & Symptoms (Select from list of complaints)	RDT	Disease Classification (Select from list of classifications)	Treatment Given (drug, dose, schedule, duration)	Referr ed?	Outco Regis d?
R10A.	…../…../…..	Age:<br>M F	Weight:<br>Temp:		Pos falc.<br>Pos viv.<br>Pos falc. & viv.<br>Neg<br>No RDT			Y N	Y
R10B.	…../…../…..	Age:<br>M F	Weight:<br>Temp:		Pos falc.<br>Pos viv.<br>Pos falc. & viv.<br>Neg<br>No RDT			Y N	Y
R10C.	…../…../…..	Age:<br>M F	Weight:<br>Temp:		Pos falc.<br>Pos viv.<br>Pos falc. & viv.<br>Neg<br>No RDT			Y N	Y
R10D.	…../…../…..	Age:<br>M F	Weight:<br>Temp:		Pos falc.<br>Pos viv.<br>Pos falc. & viv.<br>Neg<br>No RDT			Y N	Y
R10E.	…../…../…..	Age:<br>M F	Weight:<br>Temp:		Pos falc.<br>Pos viv.<br>Pos falc. & viv.<br>Neg<br>No RDT			Y N	Y

R10F.	…../…../…..	Age:<br>M F	Weight:<br>Temp:		Pos falc.<br>Pos viv.<br>Pos falc. & viv.<br>Neg<br>No RDT			Y N	Y
R10G.	…../…../…..	Age:<br>M F	Weight:<br>Temp:		Pos falc.<br>Pos viv.<br>Pos falc. & viv.<br>Neg<br>No RDT			Y N	Y
R10H.	…../…../…..	Age:<br>M F	Weight:<br>Temp:		Pos falc.<br>Pos viv.<br>Pos falc. & viv.<br>Neg<br>No RDT			Y N	Y
R10I.	…../…../…..	Age:<br>M F	Weight:<br>Temp:		Pos falc.<br>Pos viv.<br>Pos falc. & viv.<br>Neg<br>No RDT			Y N	Y
R10J.	…../…../…..	Age:<br>M F	Weight:<br>Temp:		Pos falc.<br>Pos viv.<br>Pos falc. & viv.<br>Neg<br>No RDT			Y N	Y

Ask the HEWs to answer the following questions.

R11. In the previous month, have any children been treated by the HEWs that are not recorded in the iCCM registration book?
Ji'a dabre keeysati Tajaajila qindoomina qabu sadarkaa hawaasaati kennamuun daa'imni hojjatuu Ekisteenshinii fayyaatiin gargaarsa argate fi galmee irrati kan hingalmaa'in Jira?

(1) Yes

(2) No → **Skip to HEW Questionnaire (H1)**

R12. In the previous month, how many children have been treated by the HEWs that are not recorded in the iCCM registration book?
Ji'a dabre keeyssati daa'imman meeqatuu HEFtiin tajaajila qindoomina argatanii galmee Tajaajilaa ICCM irrati hingalmoofne?

………… children (999) Don't know

R13. Why were these children not recorded in the iCCM registration book?
Maaliif daa'imman tajaajila qindoomina qabu argatan kun galmee tajaajilaa irratti galmaa'uu dhaban?

A. Treated in the community, not registered	(1) mentioned	(2) not mentioned
B. Treated in the community, other register	(1) mentioned	(2) not mentioned
C. Forgot	(1) mentioned	(2) not mentioned
D. Other, specify ……………………………………..	(1) mentioned	(2) not mentioned

END OF EQUIPMENT, SUPPLIES & SUPPORT CHECKLIST

Form 5. HEW Questionnaire

Fill out one HEW Questionnaire per HEW

Zone: …………………. Woreda: …………………. Kebele: …………………. Health post: ………………….

Data collector: ……………… **Unique HEW ID:** ☐☐☐ HEW name: ……………………

Ask the HEW the following questions:

H1. Age: ………. years

H2. What is your marital status?
Haalli ga'ela keetii maali?

(1) Married	(4) Widowed
(2) Single	(8) Other, specify …………………
(3) Separated/divorced	

H3. In what month and year did you complete the initial 1-year HEW training?
Leenjii bu'uraa HEFf kennamu bara kamii fi ji'a kam keessa xumurte?

.... month ………..year

(99) Didn't complete training

H4. In what month and year did you complete the iCCM training?
Leenjii Tajaajila qindoomina Wa'ansa da'imma sadarkaa hawaasaati yoom fudhate?

.... month ………..year

(99) Didn't complete training → ***If iCCM training not completed, skip to H6.***

H5. Did you receive a follow-up from training visit within six weeks after you received the iCCM training?
Leenjiin booda torbe 6 keessati hordoffiin siif taasifame jiraa erga leenjii tajaajila qindoomina qabu fudhate booda?

(1) Yes
(2) No
(9) Don't know
(99)NA

H6. Do you plan to continue working as an HEW through the coming year?
Bara dhufuus hojjatuu E. Fayyaa taatee hojjachuuf karoora qabdaa

(1) Yes → ***Skip to H8***
(2) No
(9) Don't know → ***Skip to H8***

H7. Why do you expect to stop working as an HEW?
Hojjatuu Ekisteenshinii fayyaa taatee hojjachuu maaliif dhaabuu yaade?

A.	Salary too low	(1) mentioned	(2) not mentioned
B.	Don't like work	(1) mentioned	(2) not mentioned
C.	Have another opportunity	(1) mentioned	(2) not mentioned
D.	Hope to receive training and move to higher level	(1) mentioned	(2) not mentioned
E.	Other, specify …………………	(1) mentioned	(2) not mentioned

H8. Where do you live now?
Amma eessa jiraatta?

(1) This kebele

(2) Other kebele → **Skip to H10**

H9. How long have you lived in this community?
Hawaasa kana keessati hammam turte?

………. months ………. years

(88) My whole life

(99) I don't live in this community

H10. How many hours did you spend doing the following activities yesterday (or last working day)?
Guyyaa kaleessaa hojiiwwan kanati aanan raawwachuuf sa'aa meeqa sijalaa fixe?
Ask the HEW to describe her activities during the day starting with when she began work.

A. Providing/offering clinical services in the health post __________

B. Providing/offering clinical services in the community __________

C. Community education/mobilization, disease prevention __________

D. Traveling outside the kebele __________

E. Other health-related activities, specify ……………………………… __________

F. Other non-health-related activities, specify ………………………… __________

G. Total work-related activities __________

H11. How many times did you visit a health center in the last 3 months?
Ji'oota 3n dabran keessa yeroo meeqa buufata fayyaa deemte?

……….. times → ***if 0 times, skip to question H14***

H12. During <u>any</u> of these visits in the last three months, did you discuss your iCCM work with a supervisor at the health center?
Buufata fayyaa yemmuu deemtu ji'oota sadeen darban kana keessati waa'ee tajaajila qindoomina qabuu fi sadarkaa hawaasaati kennamu ilaalchisee supparvaayzara kee waliin mari'atee jirtaa?

(1) Yes

(2) No → ***skip to H14***

H13. During <u>any</u> of these visits, did the supervisor do the following?
Daawwannaa kee kamuu keessati supparvaayzarri kee qabxiiwwan kanati aanan raawwateeraa?
Read list to HEW

A. Give you iCCM supplies (1) Yes (2) No (9) Don't know
Meeshaalee Tajaajila wal'aansaa qindoomina qabuuf (iCCM) oolan siif kennuu

B. Instruct you on iCCM clinical issues (1) Yes (2) No (9) Don't know
Kenniinsa tajaajila qindoomina qabu (iCCM) ilaalchisee qajeelfama ogummaa kiliinikaa siif kennuu

C. Observe you managing a sick child (1) Yes (2) No (9) Don't know
Daa'ima dhukkubsateef yemmuu tajaajila kennitu silaaluu

D. Demonstrate how to care for a sick child (1) Yes (2) No (9) Don't know
Daa'ima dhukkubsate akkamiiti akka gargaaran sigarsiisuu

E. Review clinical case scenarios (1) Yes (2) No (9) Don't know
Akkamitti akka addaan baastanii fi akka yaaltan gamaggamuu.

F. Provide verbal feedback on iCCM issues (1) Yes (2) No (9) Don't know
Dhimmoota iCCM irratti afaaniin duubdeebii siif kennuu

G. Provide written feedback on iCCM issues (1) Yes (2) No (9) Don't know
Dhimmoota iCCM irratti barreefamaan duubdeebii siif kennuu

H14. Have you ever attended an iCCM performance review and clinical mentoring meeting?

(1) Yes
(2) No

H15. What are the main reasons that caretakers refuse referral for severely ill children?
Kununisitooni tajaajila kennan daa'ima baay'ee dhukkubsate riifarii kan didaan sababoonni saan maalfaadha?

……………………………………………………………………………………………….
……………………………………………………………………………………………….
……………………………………………………………………………………………….

H16. What are the main reasons that caretakers don't bring their sick children to the health post for treatment?
Daa'ima dhukkubsate kununsiitun gara kellaa fayyaa waan hinfindneef sababni guddaan maali?

……………………………………………………………………………………………….
……………………………………………………………………………………………….
……………………………………………………………………………………………….

END OF HEW QUESTIONNAIRE

Thank the HEWs for answering your questions.
